# A Structure-Based Model for Predicting Serum Albumin Binding

**DOI:** 10.1371/journal.pone.0093323

**Published:** 2014-04-01

**Authors:** Katrina W. Lexa, Elena Dolghih, Matthew P. Jacobson

**Affiliations:** Department of Pharmaceutical Chemistry, University of California San Francisco, San Francisco, California, United States of America; Russian Academy of Sciences, Institute for Biological Instrumentation, Russian Federation

## Abstract

One of the many factors involved in determining the distribution and metabolism of a compound is the strength of its binding to human serum albumin. While experimental and QSAR approaches for determining binding to albumin exist, various factors limit their ability to provide accurate binding affinity for novel compounds. Thus, to complement the existing tools, we have developed a structure-based model of serum albumin binding. Our approach for predicting binding incorporated the inherent flexibility and promiscuity known to exist for albumin. We found that a weighted combination of the predicted logP and docking score most accurately distinguished between binders and nonbinders. This model was successfully used to predict serum albumin binding in a large test set of therapeutics that had experimental binding data.

## Introduction

Human serum albumin (HSA) is the most abundant protein in blood plasma, comprising 60% of the total protein content [1]. As a carrier protein, HSA is primarily responsible for the transport of non-esterified fatty acids, hormones, heme, and lipophillic xenobiotics through the bloodstream [2]. Binding interactions with serum albumin enable small molecules to be present at a much higher concentration in blood plasma than would otherwise be possible. In the past two decades, the clinical relevance of plasma protein binding has been debated in the literature [3], [4]. However, it is accepted that strong binding to serum proteins, particularly albumin, may be manipulated to affect pharmacokinetics and in particular the volume of distribution of the small molecule. High levels of HSA binding sequester the compound, thereby lowering the amount available to bind the target protein, but also decreasing the rate of clearance [5]. Additionally, HSA is important for passive permeability and penetration across the blood-brain barrier, as only the unbound fraction of a compound is available to diffuse out of the vasculature and into its target tissue [6]. Therefore, interaction with HSA influences the absorption, distribution, metabolism, and excretion (ADME) of small molecules [3], [7]. Optimization of the ADME profile has become integral to drug discovery programs.

**Figure 1:**
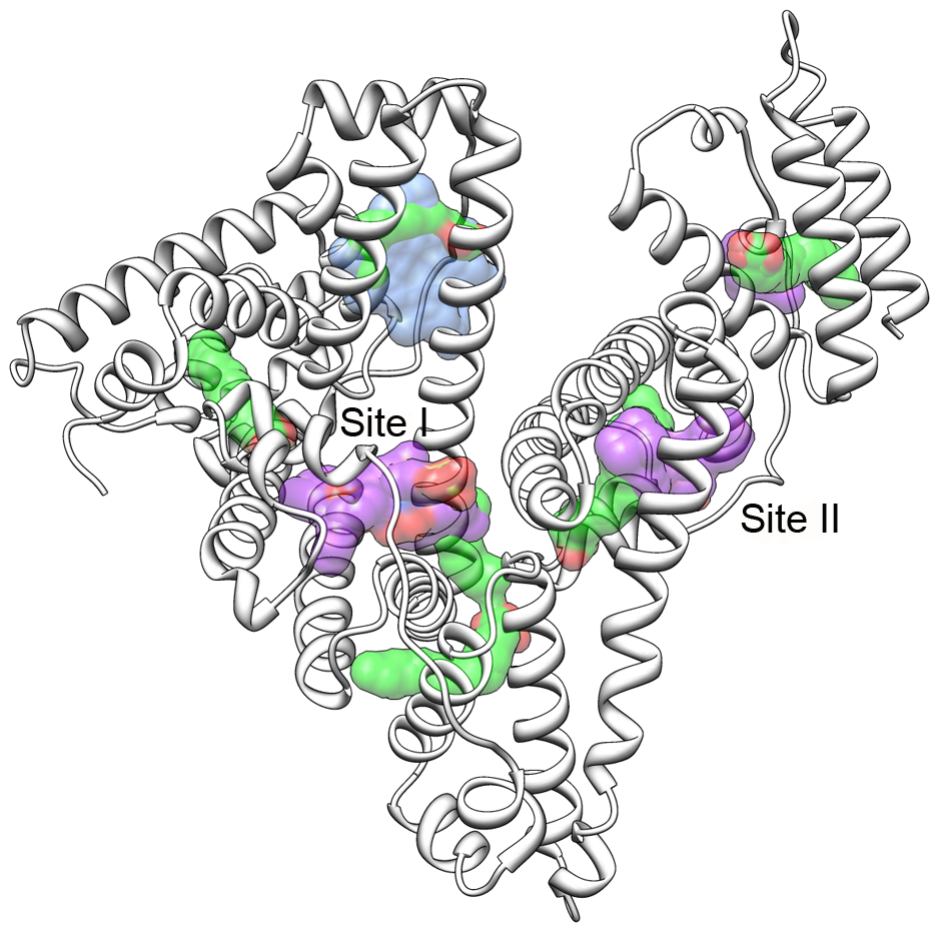
Structure of HSA with binding sites shown. The three domains comprising the structure of HSA are shown in white ribbon, with seven FA displayed in green. The pocket dimensions of sites I and II are illustrated with a purple surface, and the dimensions of site III (the heme-binding site) are shown in blue.

Here, we have developed a structural model of serum albumin binding to enable prediction of HSA binding, with a particular focus on the role of HSA conformational flexibility. HSA is a 66-kDa protein composed of 585 amino acids comprising three homologous domains, seven fatty acid (FA) binding sites, and two major structurally-selective small molecule sites (Figure 1) [8], [9]. Site I is often referred to as the warfarin site and offers primarily hydrophobic interactions to site I ligands, which are typically large, heterocyclic, and negatively charged [8]. Conversely, site II offers hydrophobic, hydrogen-bonding, and electrostatic interactions, to ligands that are usually small, aromatic, carboxylic acids. Some compounds are known to bind both sites, while other compounds can interact at additional sites on serum albumin at sufficiently high concentrations [8], [9]. Fatty acids may either compete or cooperate with small molecules for binding to HSA, and predicting the scope of their interaction with a specific ligand remains largely unachievable [1]. We aim to provide a more complete structural representation of where and how specific ligands will bind HSA, to assist in the optimization of ADME properties related to serum albumin binding.

**Figure 2:**
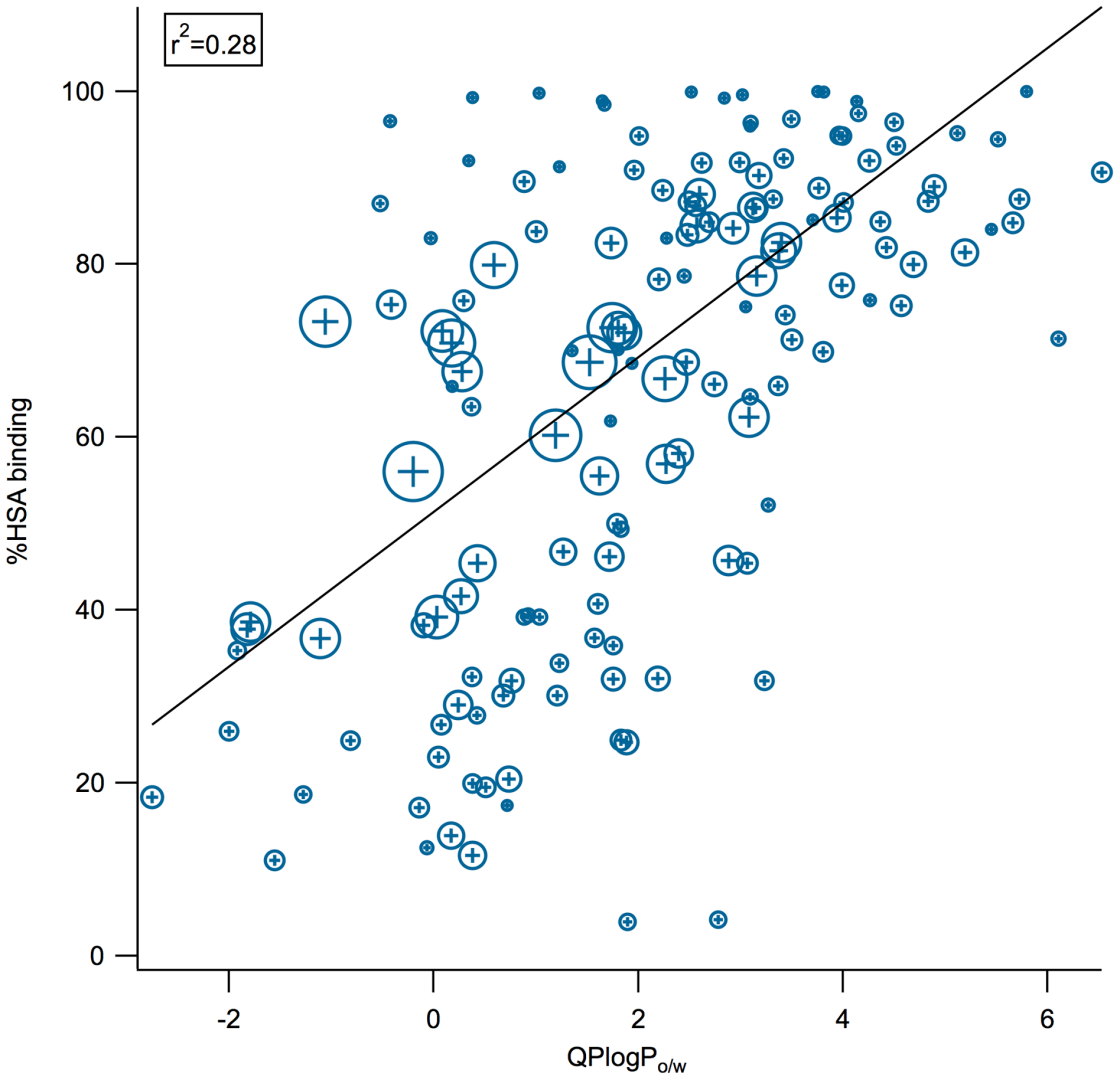
Comparison between logP and %HSA. The weak correlation between calculated logP (octanol/water) from QikProp (*QPlogP_o/w_*) and experimentally-determined %HSA binding is illustrated. In cases where data for the same compound has been reported in multiple publications, we compute a standard deviation of the reported value, represented here by marker size.

In vitro assays and quantitative structure-activity relationships (QSAR) have been used to predict small molecule binding to HSA; however, both techniques have important limitations [2], [6], [10]–[17]. HSA binding may be measured by equilibrium dialysis (the gold standard), ultrafiltration, ultracentrifugation, (fast gradient) high performance liquid chromatography, charcoal adsorption, high performance affinity chromatography (HPAC), high performance frontal analysis, solid-phase microextraction, or *in vivo* microdialysis [7], [16], [17]. These generate different measures of binding affinity, not all of which are equally precise. Difficulties in the experimental determination of HSA binding include nonspecific adsorption to the dialysis membrane [16], stability of the drug over the timescale of the experiment, sensitivity to pH, poor responsiveness to low-affinity binders, as well as expense and time requirements. Additionally, serum protein binding can be concentration-dependent and in some assays, like microdialysis and ultrafiltration, the ligand concentration changes over the course of the experiment or exists in a gradient, which must be carefully corrected for during analysis [16], [17]. Li *et al.* reviewed the published QSAR models for predicting plasma-protein binding and HSA binding, developed their own QSAR model for plasma-protein binding (r^2^ = 0.85, test set of 16 compounds), and stressed that albumin binding could not be explained by a single physiochemical property [8], [9], [18]. Indeed, while logP is a major component of all the existing quantitative models of HSA binding, a comparison of calculated logP values to %HSA values shows that lipophilicity is necessary but not sufficient to explain HSA binding (Figure 2).

QSAR and other statistical predictive models rely on the quality and size of the training set; their domain of applicability can limit their general utility. In addition, they provide little mechanistic understanding of binding relationships, including any description of the influence of FA binding. Colmenarejo *et al.* assayed HSA binding by HPAC and then applied QSAR to successfully model the HSA binding constants for 95 small molecules (r^2^ = 0.82, test set of 10 compounds) [19], [20]. Votano *et al.* applied several different modeling techniques to develop a QSAR model for plasma protein binding against a set of 1008 compounds compiled from the available literature on pharmaceutical compounds [8], [9], [21]. The authors found that an artificial neural network model performed best when predicting % plasma protein bound (r^2^ = 0.90, training set of 808 compounds; r^2^ = 0.70, test set of 200 compounds). Recently, Hall *et al.* implemented a KNIME workflow based on QSAR modeling of HSA binding affinity and site specificity [22]. Since they observed poor agreement between the data from Colmenarejo [20] and Valko [23], the authors chose to use the HPLC retention data for 120 ligands from Valko *et al* [23]. The best model of binding affinity (r^2^ = 0.68, test set of 28 compounds) included four QikProp descriptors: the number of carboxylic acids, the hydrophobic solvent-accessible surface area, the octanol/water partition coefficient, and the conformation-independent aqueous solubility. Ligands from the Kratchowil [24] set were also used to develop a naïve Bayesian classification to predict site I versus site II binding, with a reported average accuracy of 80%.

A few computational models for serum albumin have incorporated ligand docking in some manner. Zsila *et al.* performed AutoDock Vina calculations, treating HSA as rigid, to obtain 3D docking descriptors, such as binding pose geometry, with a training set of 60 small molecules [8], [25]. Those results were combined with calculated physiochemical properties to develop a support vector machine classification to predict HSA binding. Five physiochemical descriptors were selected as being critical for HSA binding, including logP, molecular weight, Ghose-Viswanadhan-Wendoloski anti-inflammatory-like index, number of carboxylic acids, and number of substituted phenyl rings. Their SVM model was 78% successful for their test set, which contained of 40 compounds. The KNIME workflow of Hall *et al.* also provides the option of performing an induced fit docking (IFD) step to predict poses [22]. They reported the results of IFD with GlideSP scoring to predict the binding pose of five site I ligands and three site II ligands; the poses with the lowest RMSD to crystal data were found within 0.82–2.75 Å. The docking scores however were not incorporated into the QSAR models.

Many challenges to the accurate structural prediction of HSA binding exist, including the moderate-to-poor resolution of the serum albumin structures deposited in the PDB and, in many cases, the poor electron density for crystallographic ligand(s). Analyses of structural data for protein-ligand binding must use high-quality information in order to provide accurate insight; a resolution better than 2.5 Å and a real-space correlation coefficient (RSCC) greater than 0.90 are useful filters when selecting crystallographic data [26]. RSCC provides a goodness-of-fit metric, based on the correlation between the map obtained from the structural model and the map calculated from the experimental data [27]. As of publication, two-thirds of the human HSA structures in the PDB have a resolution worse than 2.5 Å (average is 2.6 Å), and the ligands present in those structures have RSCC ranging from 0.82–0.95. For this reason, we exercise care in comparing computed docking poses to the reported crystallographic ligand poses [28], [29]. Furthermore, since the role of FAs in ligand-HSA binding is not well understood, they have not been included in any published calculations of HSA binding. However, since they can influence small molecule binding to HSA, they are important to a complete picture of the significant structural interactions (Figure S1) [30], [31]. Finally, many compounds are capable of binding to both sites, albeit with differing affinities, and although binding is both stereo-selective and dose-dependent, the affinities of each enantiomer are not always reported [32], [33].

Here we describe models of HSA-ligand interactions that are capable of predicting binding site preference, binding affinity, and binding pose, all of which use induced-fit docking predictions, thus providing structural hypotheses for observed trends. We expect that our model will complement similar structure-based ADME models, including those for P-glycoprotein efflux [34], interactions with hERG [35], and metabolism by cytochrome p450s [36]–[38]. Our method offers a view into the particular role and importance of the specific and non-specific interactions that are responsible for guiding HSA-ligand binding. We envision that this model could be used to rationally design changes in the strength of HSA binding for the purposes of modifying bioavailability [3].

## Materials and Methods

Ligand structures were obtained from DrugBank [39]–[41] when available; otherwise they were taken from PubChem Compound. All ligands were prepared with LigPrep 2.4 [42], where the active form of chiral compounds was retained. Both stereoisomers of xenobiotics dosed as a racemic mixture were considered. Epik 2.1 [43] was used to assign ionization states according to a target pH of 7.0±2.0. Where necessary for ligands, selenium atoms were modeled as sulfur atoms. Predicted ADME properties, including *QPlogP_o/w_ and QPlogK_hsa_*, were generated with QikProp 3.3 [44].

Recent studies in the area of structure-based drug design have emphasized the importance of protein flexibility to accurate understanding of protein-ligand interactions [29]. Here, we employed multiple crystal structures, including both apo and holo protein, with or without bound fatty acids, in order to account for protein flexibility. The ten structures of HSA with the best resolution were downloaded from the PDB [45]: 1N5U [46] (1.90 Å), 3A73 [47] (2.19 Å), 1E7A [48] (2.20 Å), 2BXH [8] (2.25 Å), 2BXP [8] (2.30 Å), 3JRY [49] (2.30 Å), 2BXA [8], (2.35 Å), 1E7B [48] (2.38 Å), 2XW0 [50] (2.40 Å), and 1HK4 [51] (2.40 Å). These crystal structures vary in their ligand content; for example, 1N5U was crystallized with two FAs bound near site II and a heme in site III, while 2BXH was defatted HSA co-crystallized with a site II ligand, and 2BXP was co-crystallized with myristic acid and a site I ligand.

PrimeX 1.7 [52] was applied to refine the experimental density and structural information prior to docking. The default PrimeX procedure was followed: the PDB structure was imported, initial R-factors were generated, the structure was split into two rigid bodies and refined in two steps, followed by simulated annealing for 1000 steps, coordinate minimization, loop refinement, optimization of hydrogen-bonding networks, B-factor refinement, ligand placement, coordinate and B-factor minimization, solvent placement, then a final coordinate and B-factor refinement. Prior to docking, all structures of HSA were prepared with the Protein Preparation Wizard [53]. Any missing side chains were rebuilt, all waters and het groups were removed, hydrogen bonds were optimized and a full structural minimization was performed. Protonation states were assigned to optimize the hydrogen-bond network; His242 in site I was doubly protonated based on known binding site interactions. Hall *et al*. noted some difficulty with their IFD results that stemmed from the different possible protonation states of this residue, but only the neutral tautomers, protonated at either N_ε_ or N_δ_, were considered [22]. In the crystal structure of apo HSA (PDB ID 1N5U), the side chain of Arg218 was oriented into site I such that it blocked ligand binding. However, this orientation overlapped with unfavorable Fo-Fc density in the crystal structure. Refinement of the electron density with PrimeX predicted a favorable position for this side chain, which was used for docking simulations.

**Figure 3:**
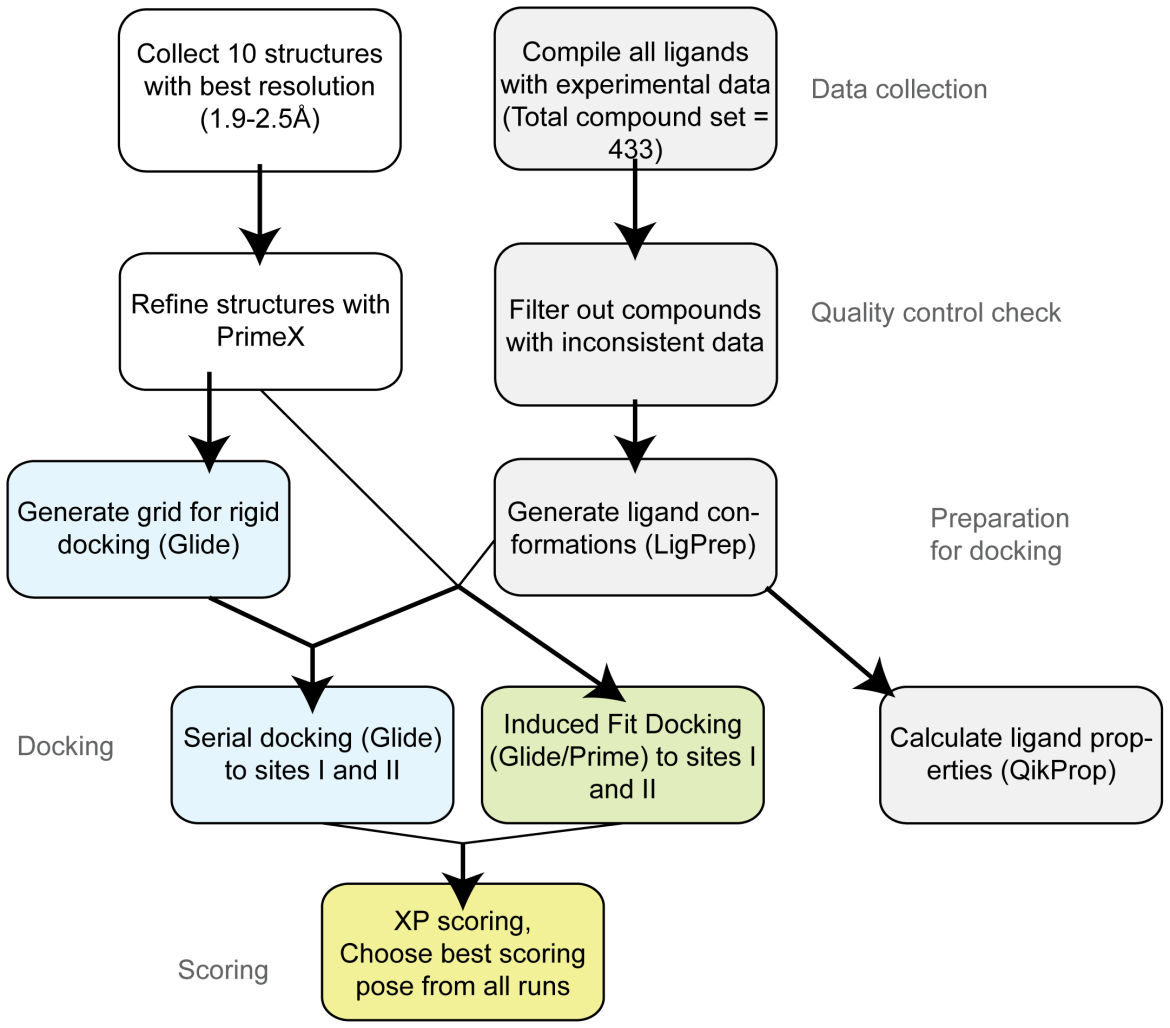
Computational workflow for docking compounds to HSA. Schematic illustrates the approach used for preparing the protein and ligand structures, docking, and analyzing the results.



An overview of the computational protocol used for predicting binding to serum albumin is illustrated in Figure 3. A set of 433 ligands with data on HSA binding was compiled from the literature. The origin of experimental data varied; therefore the analysis of our results is subdivided by data source. The model was subdivided into four datasets of diverse pharmaceutical compounds from the literature [20], [23], [25] as well as seven congeneric series [54]-[59]. In cases of review papers that collated HSA binding data, the binding affinity from the original paper reporting the data was used if discrepancies were found. In cases where data for the same compound were reported in multiple publications, the binding data was averaged, unless two or more literature values for serum albumin binding differed by more than 30% HSA bound, in which case the compound was excluded from this study (occurred for 11 compounds). The amount of a ligand that is bound to albumin (100* *f_b_*  =  %HSA) is related to Ki or K_A_ through the relationships:

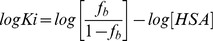

[54]



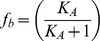

[23]

The concentration of [HSA] is assumed to be a constant value of 0.6 mM. K_A_ refers to the binding constant obtained from the compound retention time on an immobilized HSA column using affinity chromatography. The complete set of ligands and their binding classification may be found in Table S1.

Compounds were considered to be weak or nonbinders when the fraction of ligand bound to HSA (*f_b_*) was below 0.25 [21]. Literature data from binding assays, fluorescence spectroscopy, NMR, and/or crystallography established that of the 422 compounds retained, 77 are known weak/non-binders, 88 are known to preferentially bind site I, 101 are known to preferentially bind site II, and 156 are known binders but the binding site is unknown. Crystal structure information was available for 38 ligands. This dataset is larger than any previous sets of known binders and nonbinders for HSA that have been published in computational studies of HSA binding.

Rigid receptor docking with Glide [60] 5.6 was performed for all 10 crystal structures against sites I and II, resulting in a total of 20 binding predictions for each compound: we refer to the resulting predictions as the “20 site” model. Each protein structure was aligned to the best resolution crystal structure of HSA (PDB ID 1N5U). The OPLS 2001 force field was used. The rigid receptor grid was defined with an internal box of 10×10×10 Å and an external box of 30×30×30 Å. Site I was defined by the overlay of all known ligands with structural data confirming binding at that site, with a center at (30.5, 13.1, 9.7) Å when aligned to PDB ID 1N5U. Site II was defined by the centroid of all known site II ligands with available structural data; the midpoint was located at (10.25, 2.11, −13.75) Å when aligned to PDB ID 1N5U. All ligands were scored using the extra precision (XP) [61] mode.

Flexible receptor docking was performed according to the IFD workflow [62] implemented in the Schrödinger Suite 2010. The protein was subjected to a brief constrained minimization, remaining within an RMSD of 0.18 Å to the original conformation. In the preliminary round of Glide, up to three of the side chains within 5 Å of the binding site were automatically mutated to alanine (“trimmed”) based on having a residue B-factor greater than 40 Å^2^, and ligands were docked to this structure using a van der Waals scaling coefficient of 0.5. Scoring of these initial poses was done in SP mode and the best 100 poses were retained. Prime was then used to optimize all residues within 5 Å of the relevant binding site of HSA. The trimmed side chains were built back in, and surviving poses were rescored with XP. We found that the optimal results for docking into site I employed a modified version of this approach, as holding some residues fixed during minimization improved sampling of native binding positions. IFD generated poses closest to the crystallographic conformation when the only flexible residues near site I were Tyr150, Lys199, Trp214, Ala217, Arg218, Ser220, Gln221, Arg222, Asp237, His242, Cys245, Asp256, Lys262, Leu275, His288, Cys289, Val293, and Asn295. Of these, Lys199, Trp214, and His242 were mutated to Ala for the initial docking stage because they are known to be both flexible and in direct contact with the ligand.

The success of rigid receptor and induced fit docking was assessed for each binding site as well as all sites together. Correct prediction of the known binding site for ligands with site-specific experimental data was examined by comparing the ability of the model to distinguish site I binders from site II binders. The performance of all models was evaluated by comparing the percentage of known binders identified correctly (true positives) with the percentage of known weak or non-binders identified incorrectly as binders (false positives). This comparison is illustrated through the use of receiver operating characteristic (ROC) plots and analyzed according to the area under the curve (AUC) [63]. The ideal model identifies 100% true positives and 0% false positives (AUC = 1.0) and a random model finds on average 50% true positives and 50% false positives (AUC = 0.5). An AUC of 0.9 is classified as excellent, as that signifies that the structural model is capable of selecting for a true active instead of an inactive decoy 9 out of 10 times [63].

## Results and Discussion

To develop the docking strategy for HSA, we began by evaluating a variety of protocols to find the best correlation with experimental data. The major challenges are (1) the binding sites are believed to be conformationally flexible, and (2) most of the available structures have relatively poor resolution. For these reasons, the treatment of receptor flexibility in the docking protocol was our major focus. We also emphasize comparison with binding affinity data (and in particular the ability to distinguish tight binders and weak binders), because a great deal of data is available (our test set has 134 compounds); in contrast, there are only 5 PDB structures suitable for evaluating predicted binding poses (vide infra). As a point of comparison for the docking results, we also compute logP, as a simple descriptor that correlates well with HSA binding, and logK'hsa, a complex descriptor for albumin binding available in QikProp.

To establish a model capable of distinguishing high affinity binders and low/no affinity compounds, initial structural modeling was performed with a set of 134 compounds, each with experimental binding data for %HSA: 112 binders (>80% HSA binding), like warfarin and ibuprofen, and 22 weak binders (<25% HSA binding), such as penicillin V and sotalol. We refer to this set as the “strict set” throughout the remainder of the paper. The compound sets used in previous studies of HSA binding by Colmenarejo [20], Kratchowil [24], Valko [23], and Zsila [25] were combined to form the “merged set”.

It has been shown that rigid docking to multiple conformations of the same protein can be used to account for the native flexibility of a receptor when docking scores are combined [29], [64], [65]. The first docking protocol we evaluated was rigid-receptor docking, using Glide XP, against the 10 HSA structures with the best resolution. Comparison of the binding sites among these structures demonstrated significant differences in side chain conformations (which however could be due in part to poor resolution in addition to flexibility), and thus might be suitable as a representation of the intrinsic flexibility of the binding site. For each structure, we docked against both site I and site II, and chose the best scoring pose against either site. For the strict set, this protocol yielded an AUC of 0.78 in distinguishing binders from weak/non-binders. We also tested a variation in which the ligands were docked against seven known binding sites on HSA, which increased the computational expense but resulted in an AUC of 0.79, an insignificant improvement in performance relative to docking to the two main xenobiotic sites. Despite modest success at discriminating binders from weak/non-binders, the best-scoring poses generated from rigid cross-docking usually did not recover the crystallographic pose (Figure S2).

The results from rigid docking led us to hypothesize that induced fit docking (IFD) with Glide/Prime would better capture the ligand binding to HSA. The conformational changes observed with IFD were modest, within 1 Å RMSD for the backbone atoms and 3 Å RMSD for the side chains, yet we found them to be critical to the accurate categorization of HSA ligands. Conformational changes in site I were primarily observed for residues Tyr150, Lys199, Trp214, Arg218, Arg222, His288, Glu294, and in site II for Asn391, Phe403, Tyr411, Arg410, Ser489, and Arg485.

The IFD docking against site I and site II for ten different PDB structures, while computationally intensive, did not significantly improve the ability to distinguish binders and non-binders, relative to rigid docking. However, the ability to identify poses similar to those observed in crystal structures did improve (Fig S3 and Table 1), although the number of structures we can use to make this assessment is quite small (five).

**Table 1:** RMSDs of ligand heavy atoms to the crystal structure.

Ligand (PDB)	RMSD (Best XP Score)	RMSD (Best Pose)	Ligand RSCC	Ligand average B-factor	Site
IOS (2bxh)	1.52 Å (−12.33)	0.36 Å (6; −9.96)	0.987	45.0	II
cmpf (2bxa)	0.62 Å (−13.86)	0.62 Å (1; −13.86)	0.962	67.2	I
phenylbutazone (2bxp)	4.18 Å (−12.95)	1.24 Å (3; −11.34)	0.901	59.2	I
dansylphenylalanine (2xw0)	8.23 Å (−18.97)	1.01 Å (4; −15.83)	0.951	50.5	II
dansylasparagine (2xvv)	4.40 Å (−14.46)	1.50 Å (6; −12.96)	0.950	57.2	I

Root-mean-square deviation of the heavy atoms for the best scoring pose and closest RMSD pose generated from IFD, for all ligands with available crystallographic data at resolutions better than 2.5 Å and RSCC >0.90 for the ligand. The XPscore (kcal/mol) of the best RMSD pose and best scoring pose is shown in parentheses; the difference in score falls within or near the average RMSD in binding affinity of 2.3 kcal/mol for GlideXP.^61^ The rank of the ligand with the best RMSD to the crystal pose is shown in parentheses. The ligand RSCC and occupancy-weighted average B-factor enable an assessment of the reliability of the crystallographic pose.

Recent studies in structure-based drug discovery have focused on the elimination of conformer “noise” from a structural ensemble [66], [67]. In particular, it has been postulated that with adequate structural information and refinement, only a few receptor conformations are necessary to describe most binding events. While the inclusion of protein flexibility can enable a protein structure to better accommodate known binders, it may also increase the likelihood of identifying false positives [68], [69]. This can significantly impact the reliability of the model generated by flexible docking. Our results showed this to be the case, where several false positives were scored as having high affinity for HSA. In distinguishing between binders and weak binders, a simplified model consisting of IFD to site I in 2BXP and site II in 1N5U performed as well as the computationally-intensive model comprised of sites I and II in all 10 structures. The site I binding pocket in 2BXP was crystallized with phenylbutazone and myristic acid. The site II binding cavity in 1N5U was crystallized with two myristic acids bound.

Recent experimental studies have shown that binding of FAs exerted a stronger influence upon small molecules at site II compared to site I.^1^ This result is supported by the available crystal structures, which show that two FA binding sites are located in close proximity to site II, while only one exists near site I. On this basis, we performed an additional docking study where a myristic acid was retained in site II of 1N5U (Figure S4). Inclusion of the fatty acid in site II did improve the ability to distinguish between binders and weak/non-binders of HSA for both the strict and merged sets using IFD (Table 2).

**Table 2:** Predictive ability of each docking method as determined by AUC.

Docking method	Sites Docked Against	PDB structures	AUC (Strict Set)	AUC (Merged Set)
None: QPlogP_o/w_			0.92	0.78
None: QPlogKhsa			0.88	0.75
Rigid	Sites I and II	10 structures	0.78	0.68
Rigid	All 7 sites	10 structures	0.80	0.71
Rigid	Sites I and II (with fatty acid)	2BXP and 1N5U	0.68	0.64
IFD	Sites I and II	10 structures	0.78	0.69
IFD	Sites I and II	2BXP and 1N5U	0.80	0.68
IFD	Sites I and II (with fatty acid)	2BXP and 1N5U	0.83	0.74
IFD + QPlogP_o/w_	Sites I and II (with fatty acid)	2BXP and 1N5U	0.94	0.81

ROC plot AUCs are analyzed as a metric for success in predicting %HSA using *QPlogP_o/w_*, *QPlogKhsa*, rigid receptor docking, or IFD. *QPlogKhsa* is Schrödinger's QSAR-based descriptor for HSA binding and was trained on the Colmenarejo data set. The strict set is defined by all compounds with %HSA ≤25 categorized as weak/non-binders and all compounds with %HSA≥80 considered binders. The merged set encompasses the ligand sets published by Colmenarejo, Kratchowil, Valko, and Zsila for modeling HSA binding, using the same cutoff of %HSA≤25 to define weak binders. Results for each discrete set are available in Figures S5–S8 and Table S2.

**Figure 4:**
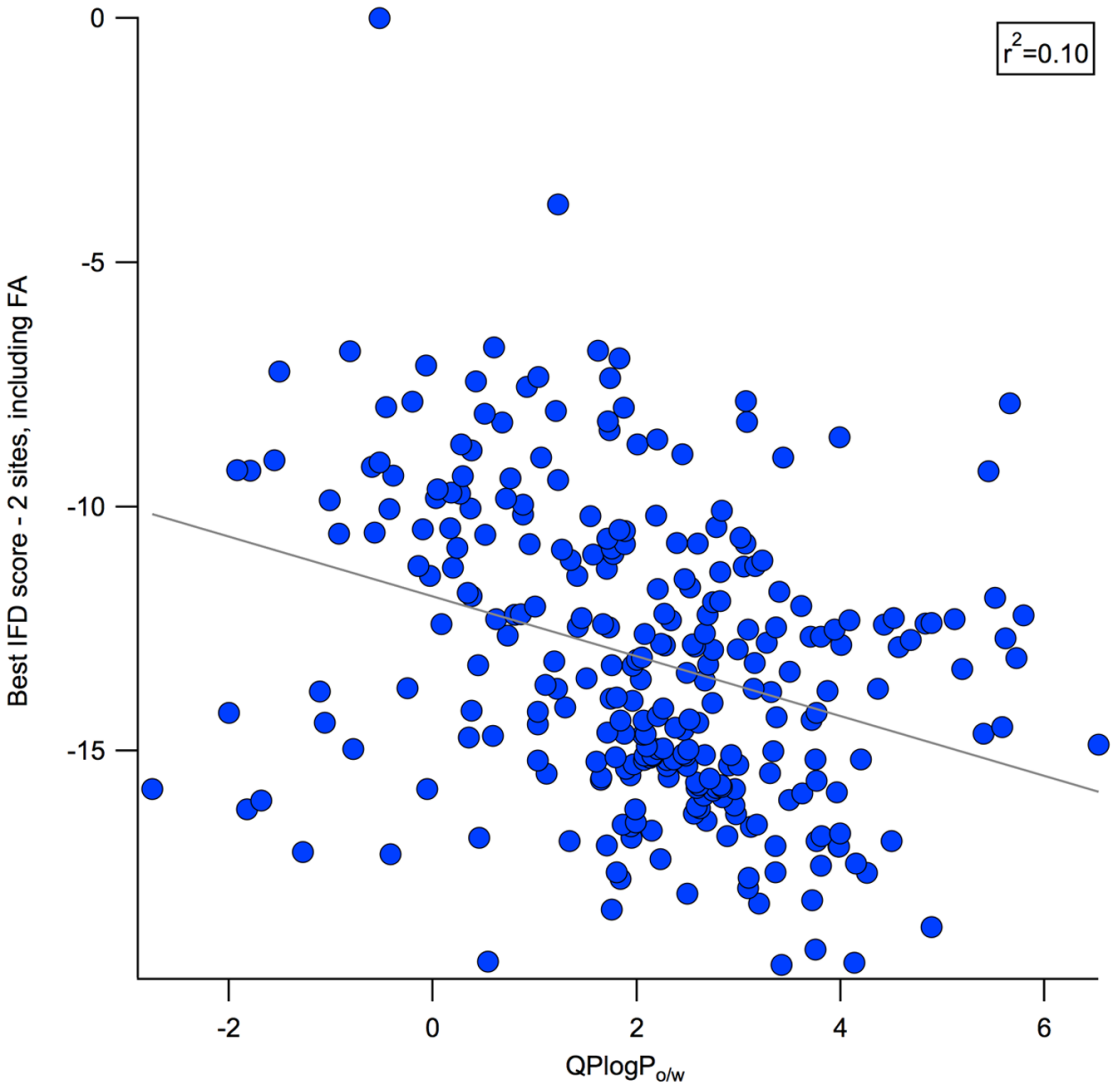
Comparison between LogP and GlideXP score. A comparison of *QPLogP_o/w_* and GlideXP scores for the strict set demonstrates that the two descriptors of HSA binding are not highly correlated with one another and therefore may be used in combination to describe the extent of serum albumin binding for a given compound.

Flexible protein-flexible ligand docking performed better than rigid docking, resulting in ligands with more favorable docking scores and poses close to experimental results. In addition, the ability to distinguish weak and strong binders was significant, with the ROC plot AUC exceeding 0.8 for the best models. However, the discriminatory power remained poorer than simply using an estimation of logP (*QPlogP_o/w_*), which is used in all QSAR studies of HSA binding, or of logK'hsa (*QPlogK_hsa_*), a QSAR-based descriptor trained on the Colmenarejo dataset. In this sense, the results of the structure-based approach were disappointing. However, we noted that the binding energy from docking did not correlate well with the calculated logP, giving an R^2^ of 0.10 (Figure 4), suggesting that the two metrics were not capturing the same information, and thus might be complementary. We thus hypothesized that an appropriate combination of the two metrics might be more predictive than either descriptor alone.

**Figure 5:**
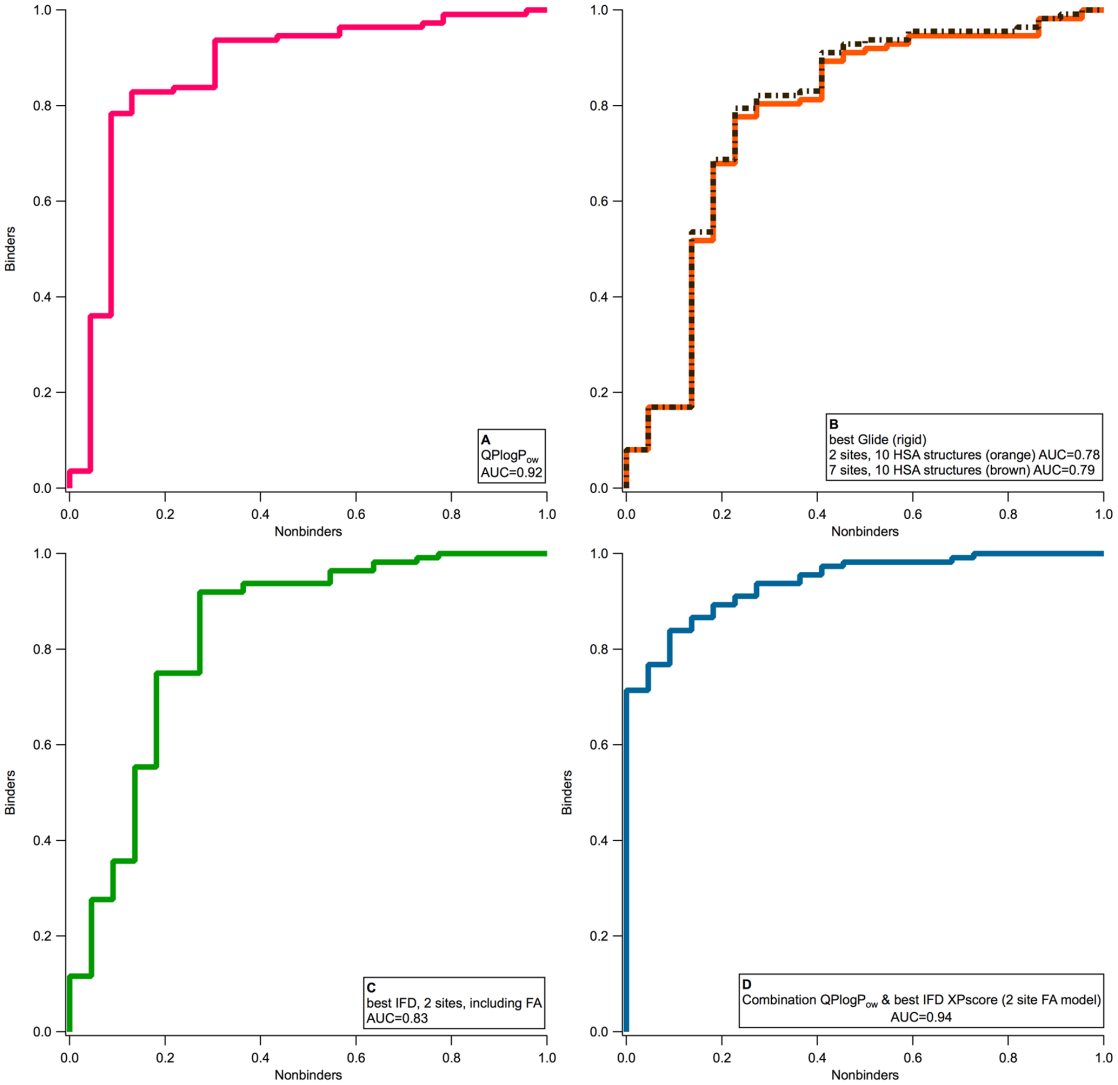
Impact of different approaches for docking the strict set to HSA. The ROC curves for the strict set of HSA binders that result from different approaches to prediction of binding affinity and pose: A) use of the calculated descriptor *QPlogP_o/w_*, B) best XP score from rigid docking with Glide to 20 sites versus 70 sites, C) best IFD score from docking to the 2 site model with a FA, and D) combined score based on *QPlogP_o/w_* and the best IFD score from the 2-site FA model.

A simple linear regression was applied to find the relationship between %HSA and the scores from IFD docking and *QPlogP_o/w_*, using bootstrapping 1000 times within the statistics package R. We found that the linear regression for %HSA binding using the *QPlogP_o/w_* and the best IFD score from the two-site FA model significantly improved our predictions of binding, giving an AUC of 0.94 for the “strict set”, using the combined score  = 25.41+α*XPscore+β*QPlogP_o/w_, where α = −1.95 and β = 7.68. The improvement relative to using the docking scores alone or the computed logP alone is particularly apparent in the early enrichment (Figure 5D); the combined model identifies >70% of the known strong binders with no false positives. The highest-ranking false positives identified by the combined model include argatroban, cromolyn, and penicillin V. The results of applying this protocol to all ligand sets available in the literature are available in the Figures S5–S8 and Table S2. The MAE files used for docking against the two-site FA model and their refinement statistics are available in the File S1, File S2, and Table S3).

**Figure 6:**
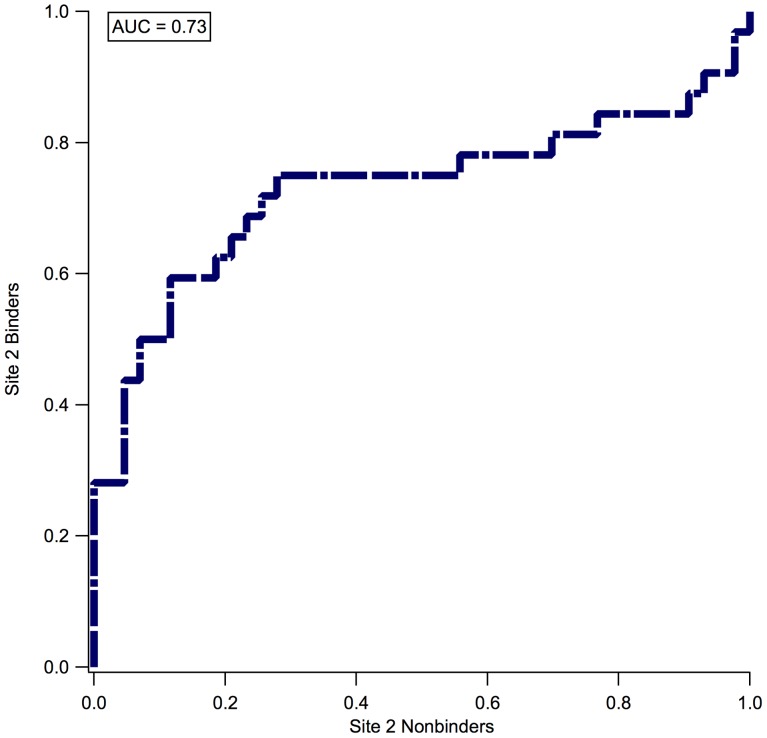
Discriminating between Site II and Site I binders. Receiver-operating characteristic curve for predicting site I binders vs. site II binders (dashed blue line).

In addition to the binding affinity of a compound for HSA, its specificity for a distinct binding site may be of interest during optimization. We examined the ability of our structural model to discriminate between binding at site I versus site II. Of the 134 ligands in our strict set, 32 are known site I ligands and 43 are known site II ligands according to published experimental data. To classify compounds by binding site, taking the difference between the docking score at site II and site I was sufficient to categorize the compound's preference for a specific binding site, with an AUC equal to 0.73 (Figure 6). A minimal difference in site I and site II docking scores was indicative of a site I binder, while a significant gap between the docking scores at site I and II corresponded with a site II binder. This can be explained by the size of the two binding pockets. Site II is known to attract small compounds, and when the binding pose is reasonable, the GlideXP terms for ligand efficiency, entropic effects, and internal energy terms tend to be more favorable for smaller ligands than for larger compounds. Using this simple analysis of the scores at each binding site enabled us to discriminate reasonably well between site I and site II binding, while simultaneously providing a reliable structural context for the binding site preference. Although rank-ordering is possible and frequently correct, our results reflect the fact that many HSA ligands can bind in a variety of positions at multiple sites along the protein surface at some high concentration, and this docking method does not account for all of those other sites.

**Figure 7:**
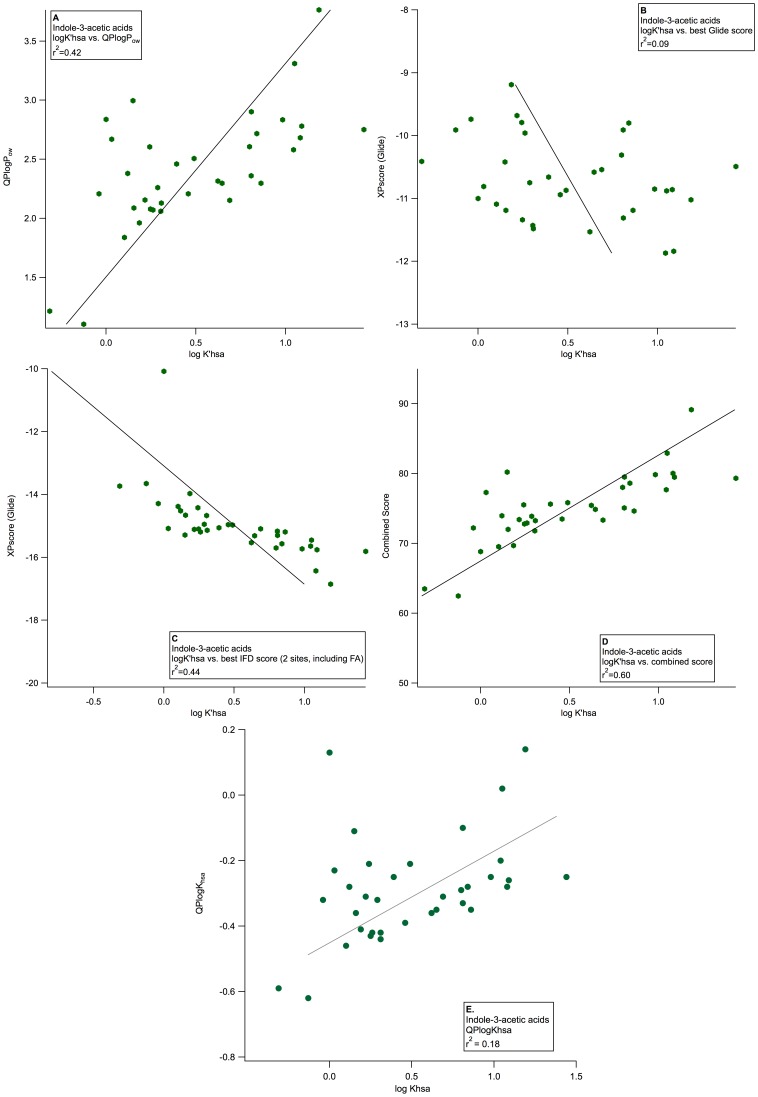
Distinguishing the impact of small structural changes on HSA binding. Predicted ranking of %HSA binding for congeneric series of indole-3-acetic acid analogues based on A) use of the calculated descriptor *QPlogP_o/w_*, B) best XP score from rigid docking with Glide to all structures, C) best IFD score from docking to the 2 site model with a FA, D) combined score based on *QPlogP_o/w_* and the best IFD score, and E) use of the QSAR-based descriptor *QPlogK_hsa_*.

While the differences in the ability of the various models to distinguish site specificity and strong versus weak binders, among chemically diverse data sets, were relatively modest, the differences in the ability to predict the impact of small structural changes upon HSA binding affinity were more striking. The application of HSA models to congeneric series is also more relevant to practical application in the context of lead optimization. Seven congeneric series with data on HSA binding affinity were available: aminoindans [55], diflunisal analogues [54], flavonoids [58], indole-3-acetic acid analogues [57], N1-alkyl pyrimidinediones [56], quinolones [59], and 2-(R)-phenylproionamides [54]. The results for the largest data set, indole-3-acetic acid analogues, are shown in Figure 7. In this case, logP correlates only modestly with HSA binding, *QPlogK_hsa_* and rigid docking scores do not correlate at all, and IFD scores show a relatively good correlation, albeit with a few major outliers. The combined logP/docking score is the most satisfactory. Those congeneric series which contained only a few data points were not as well described, as seen in the 2-(R)-phenylproionamides, but this is to be expected when the sample size and binding range is small (Figure S9). When compared to logP or XPscore alone, the combined score markedly improved the correlation between predicted and experimental HSA affinity of the congeneric series of ligands.

## Conclusion

Developing a structure-based model for HSA binding proved to be challenging. Although there are dozens of crystal structures available, most have poor resolution and/or poor electron density for the bound ligands. We expected, and the results confirmed, that treating protein flexibility would be critical to obtaining meaningful results, as judged primarily by the ability to distinguish strong vs. weak binders, or rank-ordering ligands in chemical series. However, disappointingly, it proved to be difficult to obtain results that improved relative to even the simplest and most commonly used physicochemical descriptor, logP, at least with respect to distinguishing strong and weak binders among chemically diverse ligands. Nonetheless, we argue that the available evidence suggests that the structure-based approach provides complementary information, because the docking scores correlate very weakly with logP, and a combined model using both the docking scores and logP showed good performance across a broad range of test sets. The computational cost of IFD is significant in comparison to QSAR modeling or rigid docking; therefore this approach is best applied to rigorously analyze selected compounds from an initial screen. The value of the structure-based approach seemed to be particularly clear in congeneric series, although we could only identify a few data sets with significant numbers of data points and dynamic range in the HSA binding. We speculate that the combined model may reflect physical reality in that ligands can bind specifically to (primarily) two known sites (represented explicitly by docking), but can also bind non-specifically across a number of other sites in the protein (driven by overall hydrophobicity, logP). The utility of this approach will ultimately be judged, in future work, by the ability to prospectively predict how small chemical changes will modulate HSA binding.

## Supporting Information

Figure S1 Impact of fatty acid binding on ligand position. The docking results for phenylbutazone illustrate the impact that fatty acids have upon the ligand binding position. A) In the best-scoring result from native docking to a fatty acid-bound HSA (PDB ID 2BXP), the ligand pose from both rigid (pink) and IFD (cyan) reproduces the crystallographic pose for phenylbutazone (gray). B) In the crystal structure of phenylbutazone bound to HSA in the absence of fatty acids (2BXC), the ligand adopts a different binding pose (sand) in site I. The results from cross-docking of phenylbutazone identify that position as the highest scoring and lowest RMSD outcome (orange). The cross docking score (XPscore = -9.9 kcal/mol) is slightly more favorable than the native docking score (XPscore = -9.5 kcal/mol).
(TIF)

Figure S2 Results from rigid cross-docking. Cross-docking with rigid Glide: best scoring pose (yellow) for A) dansylphenylalanine in site II (XPscore = −13.0 kcal/mol; PDB ID 2BXP), B) iophenoxic acid in site I (XPscore = −10.93 kcal/mol; PDB ID 2BXA), C) diflunisal in site II (XPscore = −11.9 kcal/mol; PDB ID 2BXP), and D) S-warfarin in site I (XPscore = −10.1 kcal/mol; PDB ID 2BXP) overlaid with the crystallographic position of the ligand (gray) from PDB ID 2XW0, 2YDF, 2BXE, and 1HA2 respectively.
(TIF)

Figure S3 Results from induced fit cross-docking. Cross-docking with IFD: best scoring pose (green) for A) dansylphenylalanine in site II (XPscore = −18.97 kcal/mol; PDB ID 1N5U), B) iophenoxic acid in site I (XPscore = −13.19 kcal/mol; PDB ID 2BXH), C) diflunisal in site II (XPscore = −17.88 kcal/mol; PDB ID 1N5U), and D) S-warfarin in site I (XPscore = −12.81 kcal/mol; PDB ID 2BXP) overlaid with the crystallographic position of the ligand (gray) from PDB ID 2XW0, 2YDF, 2BXE, and 1HA2 respectively.
(TIF)

Figure S4 Position of myristic acid in site II. Myristic acid residue 1003 in site II of 1N5U.
(TIF)

Figure S5 Results for Colmenarejo set. A comparison of the ROC curves for the Colmenarejo set of HSA binders that result from different approaches to prediction of binding affinity and pose: A) use of the calculated descriptor *QPlogP_o/w_*, B) best XP score from rigid docking with Glide to all structures, C) best IFD score from docking to the 2 site model with a FA, and D) combined score based on *QPlogP_o/w_* and the best IFD score from the 2-site FA model.
(TIF)

Figure S6 Results for Kratchowil set. A comparison of the ROC curves for the Kratchowil set of HSA binders that result from different approaches to prediction of binding affinity and pose: A) use of the calculated descriptor *QPlogP_o/w_*, B) best XP score from rigid docking with Glide to all structures, C) best IFD score from docking to the 2 site model with a FA, and D) combined score based on *QPlogP_o/w_* and the best IFD score from the 2-site FA model.
(TIF)

Figure S7 Results for Valko set. A comparison of the ROC curves for the Valko set of HSA binders that result from different approaches to prediction of binding affinity and pose: A) use of the calculated descriptor *QPlogP_o/w_*, B) best XP score from rigid docking with Glide to all structures, C) best IFD score from docking to the 2 site model with a FA, and D) combined score based on *QPlogP_o/w_* and the best IFD score from the 2-site FA model.
(TIF)

Figure S8 Results for Zsila set. A comparison of the ROC curves for the Zsila set of HSA binders that result from different approaches to prediction of binding affinity and pose: A) use of the calculated descriptor *QPlogP_o/w_*, B) best XP score from rigid docking with Glide to all structures, C) best IFD score from docking to the 2 site model with a FA, and D) combined score based on *QPlogP_o/w_* and the best IFD score from the 2-site FA model.
(TIF)

Figure S9 Predicting HSA binding for distinct congeneric series. Correlation between combined score (XPscore from IFD and *QPlogP_o/w_*) and experimental value for HSA binding for A) aminoindan series, B) flavonoids, C) N1-alkyl pyrimidinedione series, D) quinolines, E) 2-(R) phenylproionamides, and F) diflunisals.
(TIF)

Table S1 Ligands used for study, including binder type and average HSA score. All ligands collected for the study, with their status as an HSA binder described as 0 = binder, 1 = nonbinder, and 2 = unclear from data. Their average %HSA score and SD if applicable are also shown.
(DOCX)

Table S2 Results from model applied to four literature sets. Literature data sets for HSA binding, derived from Colmenarejo, Kratchowil, Valko, and Zsila, as well as all ligands with binder/nonbinder status. ROC plot AUCs are analyzed as a metric for success in predicting binding to HSA using *QPlogP_o/w_*; Schrödinger's metric for HSA binding, *QPlogKhsa*, which was developed using the Colmenarejo set and overlaps with the Valko set; rigid receptor docking; or IFD. The Colmenarejo set includes high-scoring false positives cromolyn, ebselen, and pencillin V.
(DOCX)

Table S3 **Refinement scores for PDB structures.** PrimeX refinement scores for the two PDB structures used in the final, combined, model.
(DOCX)

File S1 MAE file used for docking to site I.
(MAE)

File S2 MAE file used for docking to site II.
(MAE)
