# Supplementary figures and images for: A Structure-Based Model for Predicting Serum Albumin Binding
Source: PLoS One. 2014 Apr 1;9(4):e93323. doi: 10.1371/journal.pone.0093323 (PMC3972100; doi:10.1371/journal.pone.0093323)

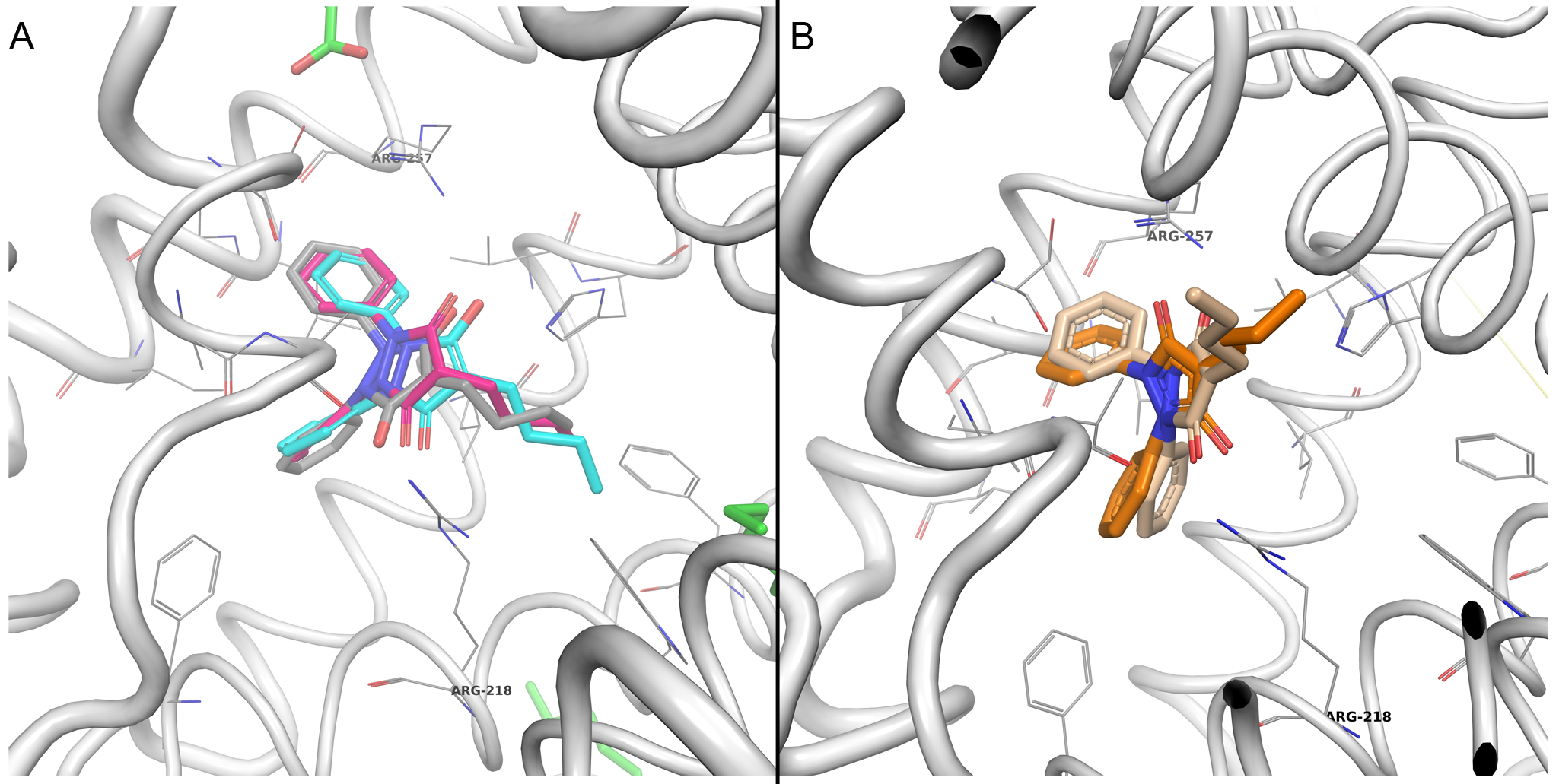

Supplement: Figure S1 — Impact of fatty acid binding on ligand position. The docking results for phenylbutazone illustrate the impact that fatty acids have upon the ligand binding position. A) In the best-scoring result from native docking to a fatty acid-bound HSA (PDB ID 2BXP), the ligand pose from both rigid (pink) and IFD (cyan) reproduces the crystallographic pose for phenylbutazone (gray). B) In the crystal structure of phenylbutazone bound to HSA in the absence of fatty acids (2BXC), the ligand adopts a different binding pose (sand) in site I. The results from cross-docking of phenylbutazone identify that position as the highest scoring and lowest RMSD outcome (orange). The cross docking score (XPscore = -9.9 kcal/mol) is slightly more favorable than the native docking score (XPscore = -9.5 kcal/mol). (TIF) [file pone.0093323.s001.tif]

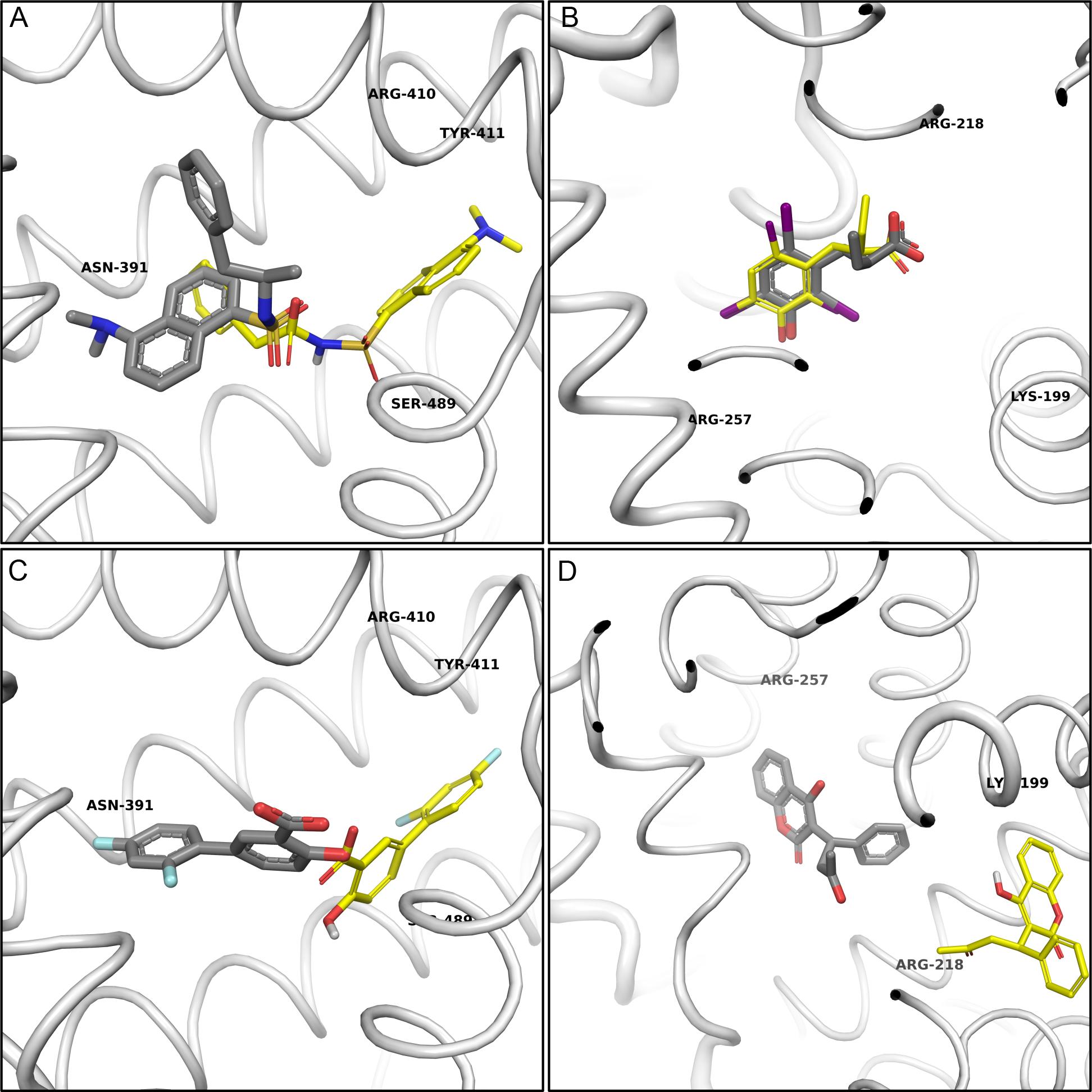

Supplement: Figure S2 — Results from rigid cross-docking. Cross-docking with rigid Glide: best scoring pose (yellow) for A) dansylphenylalanine in site II (XPscore = −13.0 kcal/mol; PDB ID 2BXP), B) iophenoxic acid in site I (XPscore = −10.93 kcal/mol; PDB ID 2BXA), C) diflunisal in site II (XPscore = −11.9 kcal/mol; PDB ID 2BXP), and D) S-warfarin in site I (XPscore = −10.1 kcal/mol; PDB ID 2BXP) overlaid with the crystallographic position of the ligand (gray) from PDB ID 2XW0, 2YDF, 2BXE, and 1HA2 respectively. (TIF) [file pone.0093323.s002.tif]

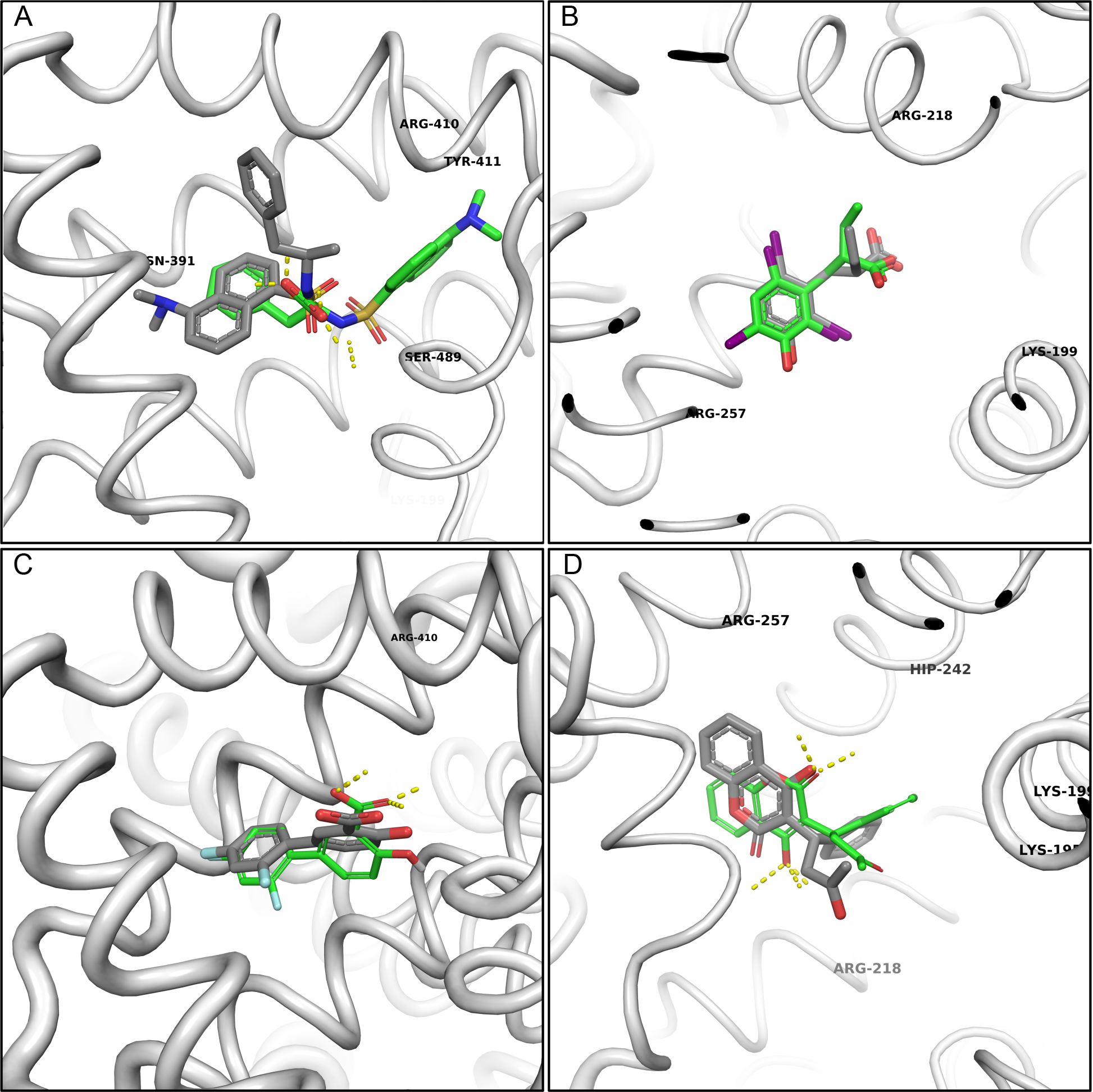

Supplement: Figure S3 — Results from induced fit cross-docking. Cross-docking with IFD: best scoring pose (green) for A) dansylphenylalanine in site II (XPscore = −18.97 kcal/mol; PDB ID 1N5U), B) iophenoxic acid in site I (XPscore = −13.19 kcal/mol; PDB ID 2BXH), C) diflunisal in site II (XPscore = −17.88 kcal/mol; PDB ID 1N5U), and D) S-warfarin in site I (XPscore = −12.81 kcal/mol; PDB ID 2BXP) overlaid with the crystallographic position of the ligand (gray) from PDB ID 2XW0, 2YDF, 2BXE, and 1HA2 respectively. (TIF) [file pone.0093323.s003.tif]

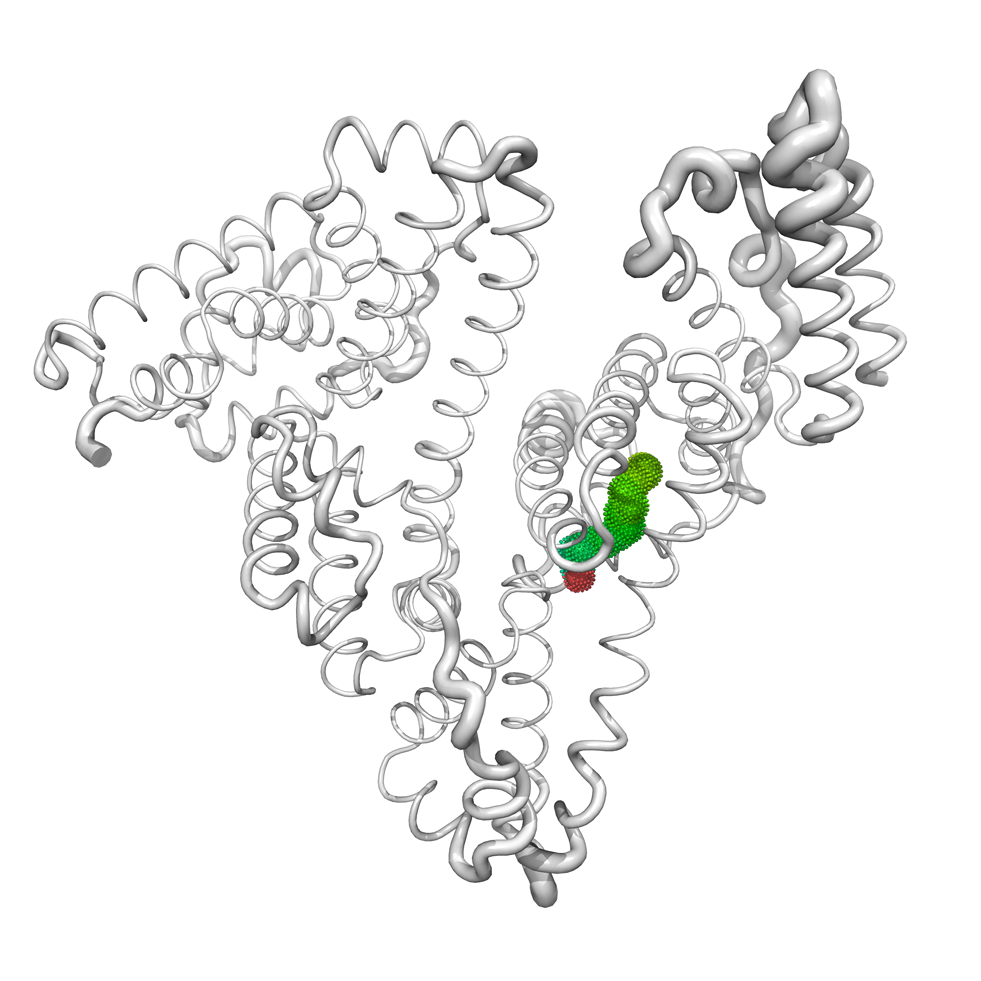

Supplement: Figure S4 — Position of myristic acid in site II. Myristic acid residue 1003 in site II of 1N5U. (TIF) [file pone.0093323.s004.tif]

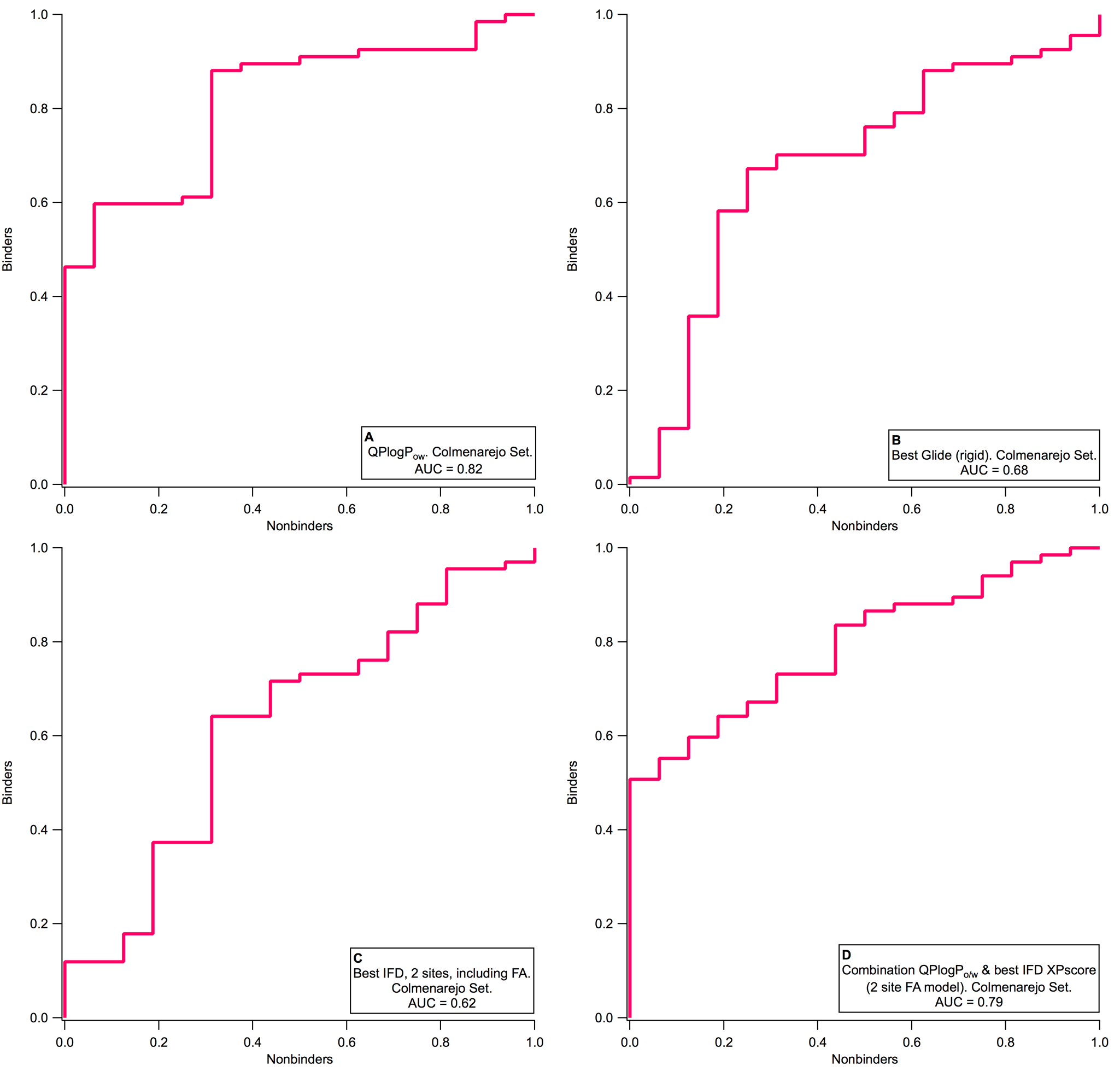

Supplement: Figure S5 — Results for Colmenarejo set. A comparison of the ROC curves for the Colmenarejo set of HSA binders that result from different approaches to prediction of binding affinity and pose: A) use of the calculated descriptor QPlogPo/w, B) best XP score from rigid docking with Glide to all structures, C) best IFD score from docking to the 2 site model with a FA, and D) combined score based on QPlogPo/w and the best IFD score from the 2-site FA model. (TIF) [file pone.0093323.s005.tif]

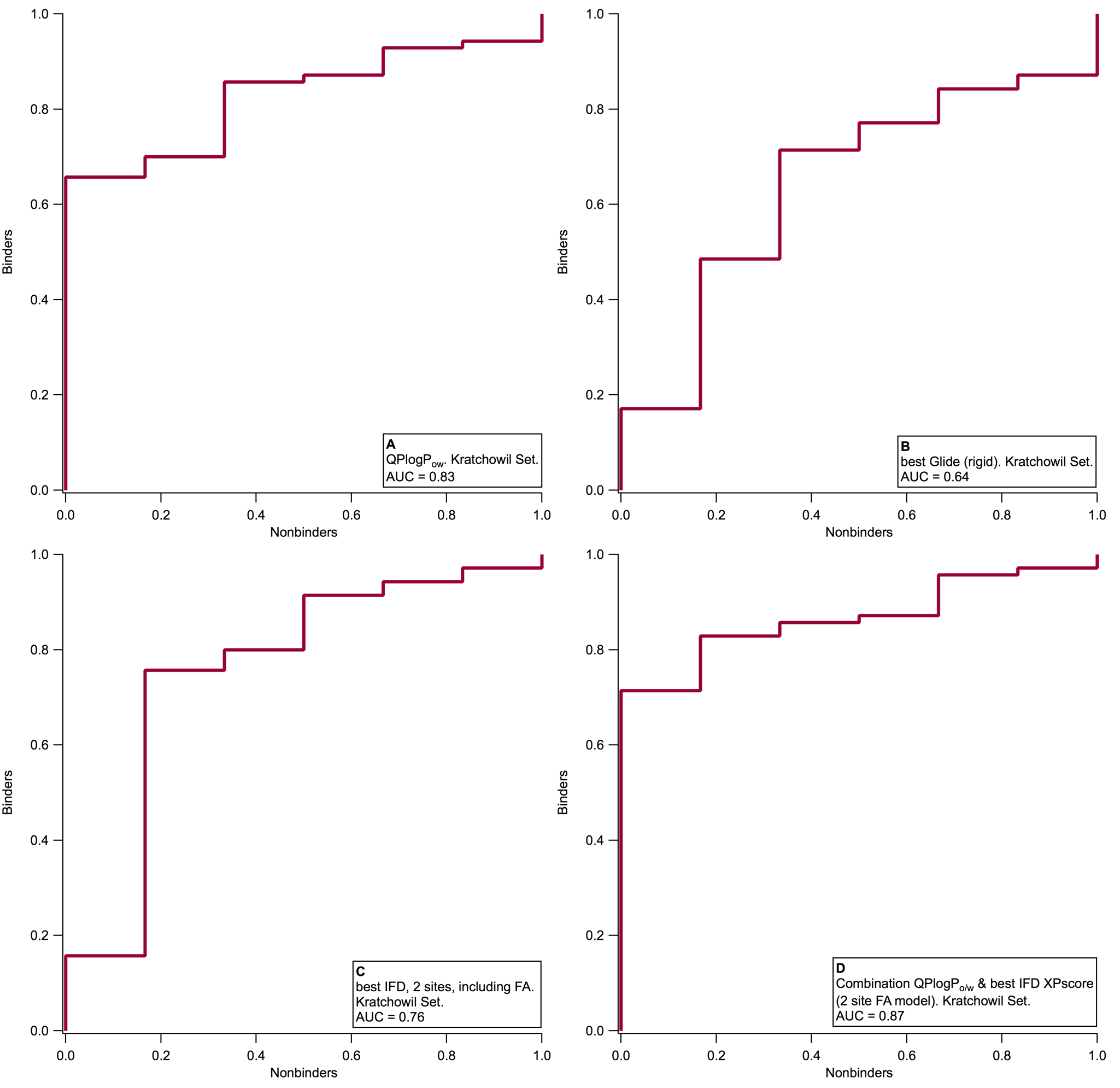

Supplement: Figure S6 — Results for Kratchowil set. A comparison of the ROC curves for the Kratchowil set of HSA binders that result from different approaches to prediction of binding affinity and pose: A) use of the calculated descriptor QPlogPo/w, B) best XP score from rigid docking with Glide to all structures, C) best IFD score from docking to the 2 site model with a FA, and D) combined score based on QPlogPo/w and the best IFD score from the 2-site FA model. (TIF) [file pone.0093323.s006.tif]

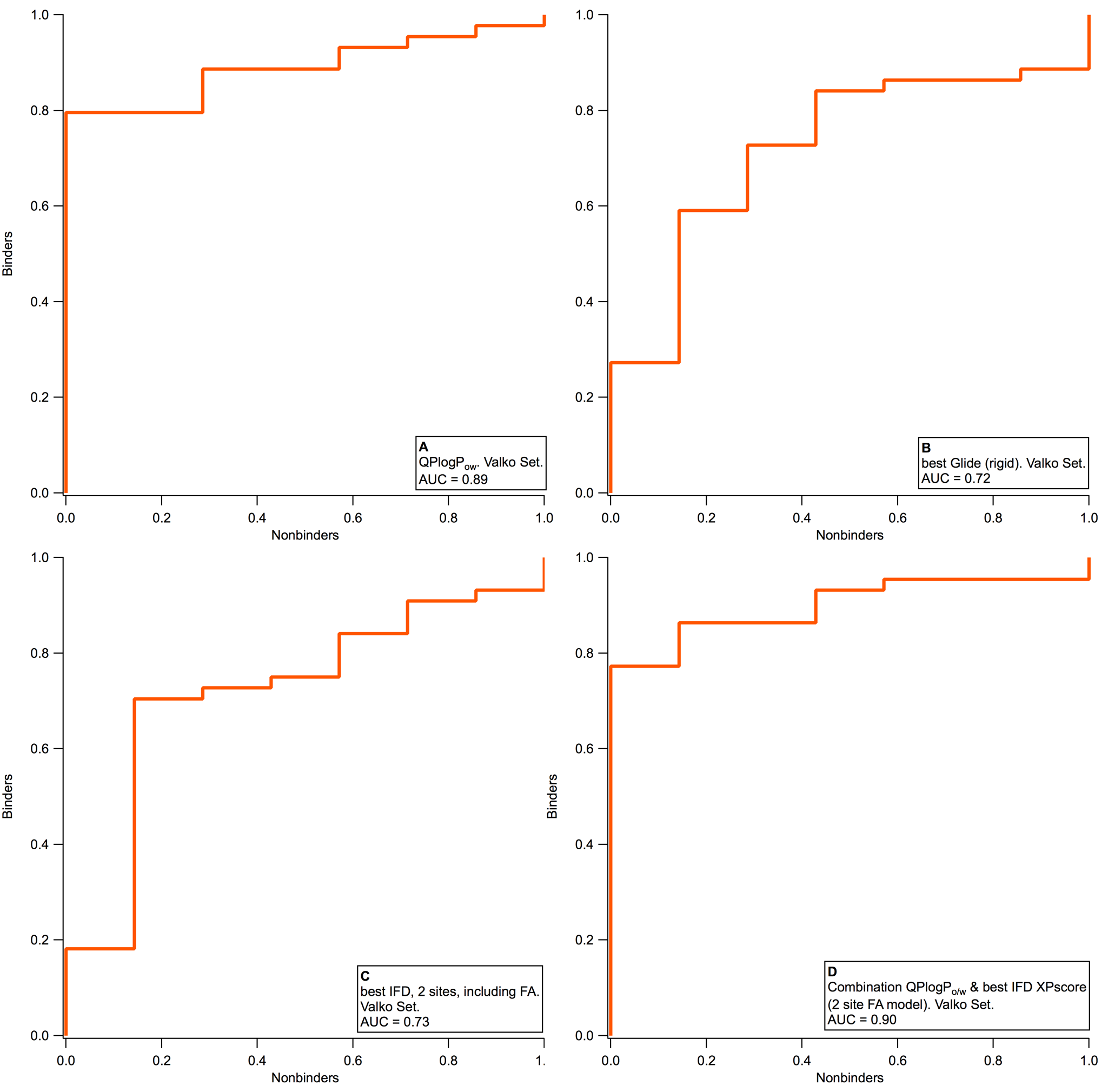

Supplement: Figure S7 — Results for Valko set. A comparison of the ROC curves for the Valko set of HSA binders that result from different approaches to prediction of binding affinity and pose: A) use of the calculated descriptor QPlogPo/w, B) best XP score from rigid docking with Glide to all structures, C) best IFD score from docking to the 2 site model with a FA, and D) combined score based on QPlogPo/w and the best IFD score from the 2-site FA model. (TIF) [file pone.0093323.s007.tif]

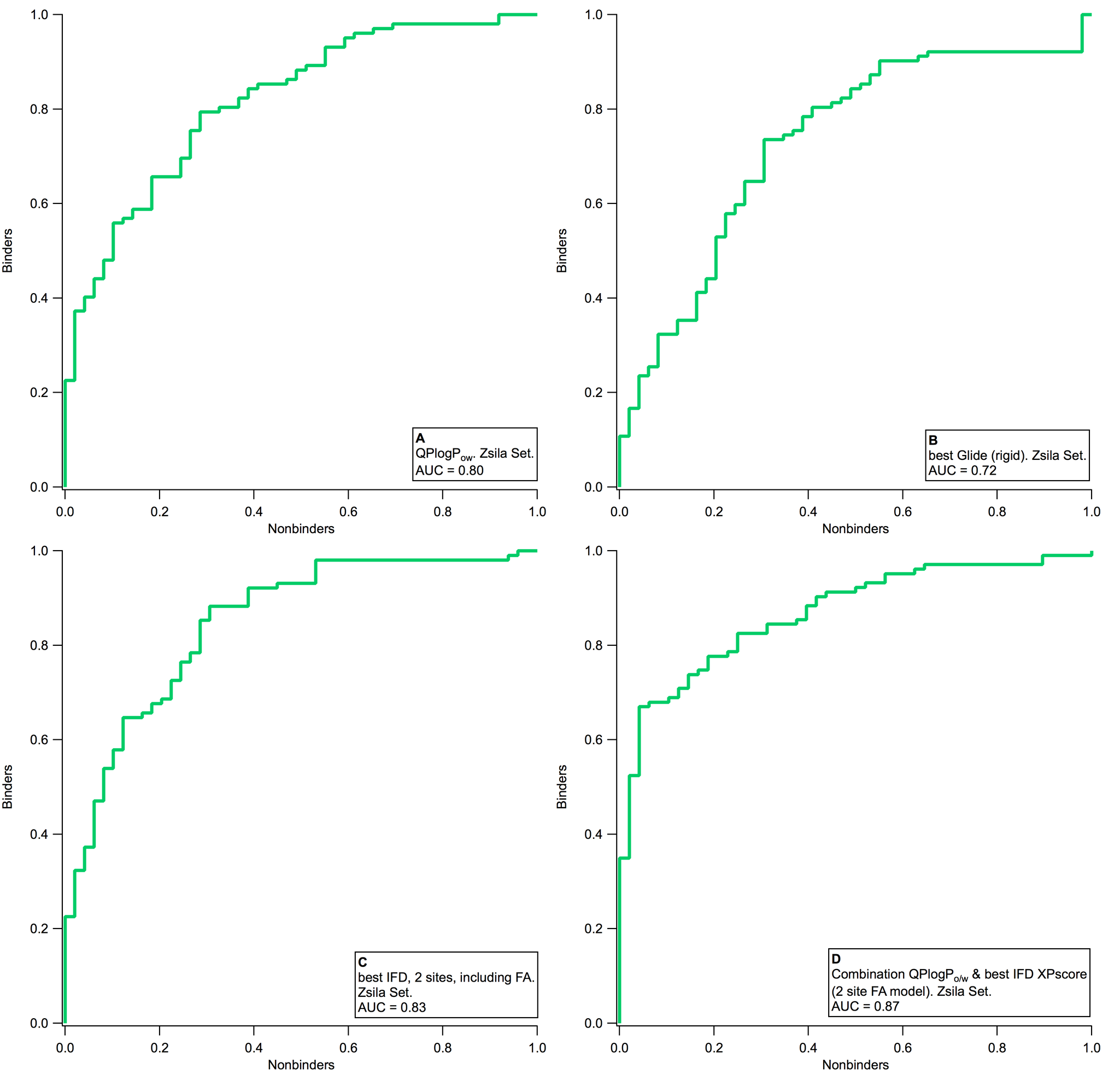

Supplement: Figure S8 — Results for Zsila set. A comparison of the ROC curves for the Zsila set of HSA binders that result from different approaches to prediction of binding affinity and pose: A) use of the calculated descriptor QPlogPo/w, B) best XP score from rigid docking with Glide to all structures, C) best IFD score from docking to the 2 site model with a FA, and D) combined score based on QPlogPo/w and the best IFD score from the 2-site FA model. (TIF) [file pone.0093323.s008.tif]

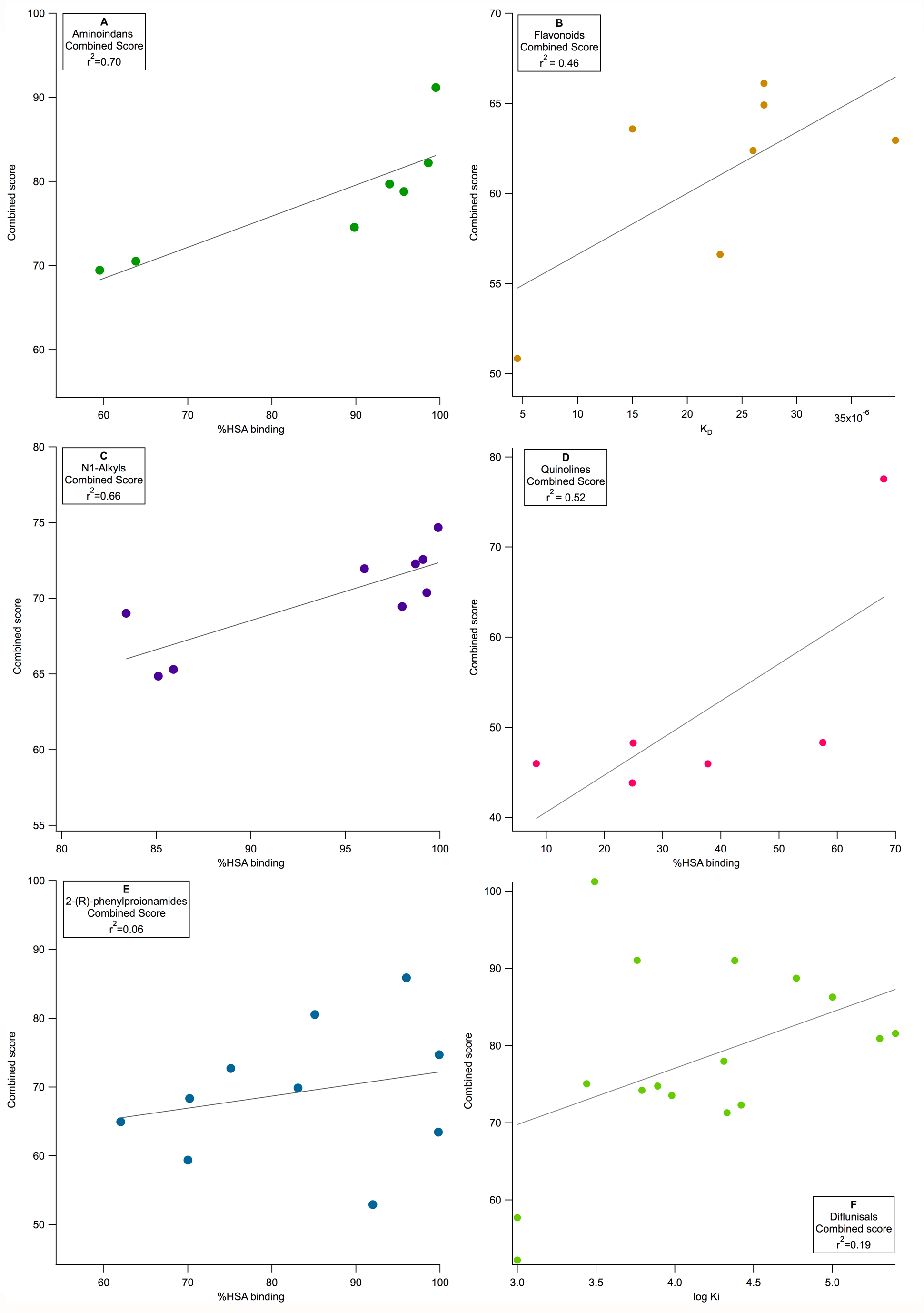

Supplement: Figure S9 — Predicting HSA binding for distinct congeneric series. Correlation between combined score (XPscore from IFD and QPlogPo/w) and experimental value for HSA binding for A) aminoindan series, B) flavonoids, C) N1-alkyl pyrimidinedione series, D) quinolines, E) 2-(R) phenylproionamides, and F) diflunisals. (TIF) [file pone.0093323.s009.tif]
