# Supplementary material for: A Structure-Based Model for Predicting Serum Albumin Binding
Source: PLoS One. 2014 Apr 1;9(4):e93323. doi: 10.1371/journal.pone.0093323 (PMC3972100; doi:10.1371/journal.pone.0093323)
Supplement: Table S1 — Ligands used for study, including binder type and average HSA score. All ligands collected for the study, with their status as an HSA binder described as 0 = binder, 1 = nonbinder, and 2 = unclear from data. Their average %HSA score and SD if applicable are also shown. (DOCX) [file pone.0093323.s010.docx]

**SI Table S1**. **Ligands used for study, including binder type and average HSA score.**

| **ligand** | **Binder/**  **Nonbinder** | **Average HSA score** | **SD HSA score** |
| --- | --- | --- | --- |
| 2-(R)-phenylproionamides 1 | 0 | 99.80 | 0.00 |
| 2-(R)-phenylproionamides 10 | 0 | 70.10 | 0.14 |
| 2-(R)-phenylproionamides 2 (Reparixin) | 0 | 99.90 | 0.00 |
| 2-(R)-phenylproionamides 3 | 0 | 91.98 | 0.03 |
| 2-(R)-phenylproionamides 4 | 0 | 70.00 | 0.01 |
| 2-(R)-phenylproionamides 5 | 0 | 61.88 | 0.17 |
| 2-(R)-phenylproionamides 6 | 0 | 85.12 | 0.03 |
| 2-(R)-phenylproionamides 7 | 0 | 75.07 | 0.04 |
| 2-(R)-phenylproionamides 8 | 0 | 83.04 | 0.08 |
| 2-(R)-phenylproionamides 9 | 0 | 95.99 | 0.01 |
| 2-furan-propionic acid | 0 |  |  |
| 3-acetylcoumarin | 0 | 93.64 |  |
| 3,5-di-iodosalicyclic acid | 0 |  |  |
| 4-chromanol | 0 | 97.59 |  |
| 4-hydroxycoumarin | 0 | 99.62 |  |
| 4-hydroxylmethyl-6-methoxy-quinolone | 0 | 73.00 |  |
| 4,7-daidzein disulfate | 0 | 98.62 |  |
| 4-daidzein sulfate | 0 | 92.34 |  |
| 6-methoxy-quinolone | 0 |  |  |
| 6-N-benzyl-2-thiouracil | 0 |  |  |
| 6,3-dihydroxydaidzein | 0 | 95.15 |  |
| 7-daidzein sulfate | 0 | 94.43 |  |
| 8,3-dihydroxydaidzein | 0 | 96.62 |  |
| 8-hydroxydaidzein | 0 | 94.43 |  |
| Abacavir | 0 | 91.25 | 0.35 |
| Acebutolol | 0 | 38.14 |  |
| Acrivastin | 0 | 48.85 |  |
| Acrylodan | 0 |  |  |
| Aformoterol | 0 | 31.70 |  |
| Albendazole | 0 |  |  |
| Alendronate | 0 |  |  |
| Alfentanil | 0 |  |  |
| Alfuzosin | 0 |  |  |
| Alprazolam | 0 | 80.00 |  |
| Alprenolol | 0 | 45.44 | 9.70 |
| Aminodarone | 0 |  |  |
| Aminoindan Analogue 1a | 0 | 63.80 |  |
| Aminoindan Analogue 1b | 0 | 89.80 |  |
| Aminoindan Analogue 1c (Indacaterol) | 0 | 95.70 |  |
| Aminoindan Analogue 1d | 0 | 98.60 |  |
| Aminoindan Analogue 1e | 0 | 99.50 |  |
| Aminoindan Analogue 1f | 0 | 59.50 |  |
| Aminoindan Analogue 1g | 0 | 94.00 |  |
| Amphitericin B | 0 |  |  |
| Ampicillin | 0 | 25.97 | 7.41 |
| Amrinone | 0 | 65.84 | 0.37 |
| Anastrozole | 0 |  |  |
| Aripiprazine | 0 |  |  |
| Atazanavir | 0 | 84.00 | 0.00 |
| Atorvastatin | 0 |  |  |
| Atovaquone | 0 | 99.90 |  |
| Azapropazone | 0 | 99.59 | 0.26 |
| Azidocillin | 0 |  |  |
| Aztreonam | 0 |  |  |
| Baicalein | 0 |  |  |
| BCA | 0 |  |  |
| Beclomethasone | 0 | 68.52 | 0.54 |
| Benoxaprofen | 0 | 99.91 |  |
| Betamethasone | 0 | 49.35 | 4.57 |
| Bonomycin | 0 | 61.86 |  |
| Bromazepam | 0 | 46.20 | 17.45 |
| Budesonide | 0 | 78.23 | 10.58 |
| Bumetanide | 0 | 72.59 | 21.58 |
| Bupropion | 0 | 47.12 |  |
| Calcitriol | 0 | 97.35 |  |
| Camptothecin | 0 | 72.68 | 38.57 |
| Carbamazepine | 0 | 58.09 | 18.17 |
| Carmoterol | 0 | 51.70 |  |
| Carprofen | 0 | 99.90 | 0.09 |
| Cefaclor | 0 | 7.17 |  |
| Cefadroxil | 0 | 53.34 |  |
| Cefazolin | 0 | 75.33 | 17.82 |
| Ceftazidime | 0 | 17.13 | 8.52 |
| Celecoxib | 0 | 96.79 |  |
| Cephalexin | 0 | 36.71 | 29.03 |
| CFL (2‘Fluoro-2’-Deoxy-Cytidine-5’-Monophosphate) | 0 |  |  |
| Chloramphenicol | 0 | 25.75 |  |
| Chlorazepate | 0 |  |  |
| Chlorodiazepoxide | 0 |  |  |
| Chlorothiazide | 0 | 96.56 | 0.70 |
| Chlorpromazine | 0 | 95.17 | 2.79 |
| Chlorpropamide | 0 | 78.65 | 28.29 |
| Chlorthalidone | 0 | 68.00 |  |
| Chromone-2-Carboxylic acid | 0 |  |  |
| Cimetidine | 0 | 27.79 | 6.03 |
| Ciprofloxacin | 0 | 67.58 | 28.61 |
| Clofibrate | 0 | 88.11 | 19.97 |
| Clomipramine | 0 |  |  |
| Clonidine | 0 | 36.78 | 7.69 |
| Clotrimazole | 0 | 95.63 |  |
| Cloxacillin | 0 | 83.40 | 10.74 |
| CMPF (3-carboxy-4-methyl-5-propyl-2-furanpropanoic acid) | 0 | 99.99 |  |
| Compound 1 | 0 |  |  |
| Compound 2 | 0 |  |  |
| Creosol | 0 |  |  |
| Cyclosporine | 0 |  |  |
| Cytarabine | 0 | 35.31 | 6.37 |
| Daidzein | 0 |  |  |
| Daidzein | 0 | 94.61 |  |
| Dansyl-L-amide | 0 |  |  |
| Dansyl-L-arginine | 0 |  |  |
| Dansyl-L-asparagine | 0 | 98.91 |  |
| Dansyl-L-glutamate | 0 |  |  |
| Dansyl-L-glycine | 0 | 35.46 |  |
| Dansyl-L-norvaline | 0 |  |  |
| Dansyl-L-phenylalanine | 0 | 99.63 |  |
| Dansyl-L-proline | 0 |  |  |
| Dansyl-L-sarcosine | 0 | 99.66 |  |
| Dapsone | 0 |  |  |
| Darunavir | 0 |  |  |
| Desipramine | 0 | 89.00 | 12.31 |
| Diazepam | 0 | 91.78 | 9.10 |
| Diclofenac | 0 | 96.40 | 6.10 |
| Dicloxacillin | 0 | 86.74 | 10.35 |
| Dicoumarol | 0 | 99.87 |  |
| Didanosine | 0 | 87.00 | 4.24 |
| Diflunisal | 0 | 94.90 | 6.73 |
| Diflunisal analogues 12 | 0 | 98.36 |  |
| Diflunisal analogues 14 | 0 | 94.04 |  |
| Diflunisal analogues 15 | 0 | 92.45 |  |
| Diflunisal analogues 16 (5-chloro-salicylic acid) | 0 | 92.77 |  |
| Diflunisal analogues 17 | 0 | 85.14 |  |
| Diflunisal analogues 18 | 0 | 99.34 |  |
| Diflunisal analogues 19 | 0 | 99.17 |  |
| Diflunisal analogues 20 | 0 | 78.72 |  |
| Diflunisal analogues 21 | 0 | 82.32 |  |
| Diflunisal analogues 22 | 0 | 37.50 |  |
| Diflunisal analogues 23 | 0 | 37.50 |  |
| Diflunisal analogues 24 | 0 | 77.54 |  |
| Diflunisal analogues 25 | 0 | 64.96 |  |
| Diflunisal analogues 26 | 0 | 93.50 |  |
| Diflunisal analogues 27 | 0 | 62.30 |  |
| Digitoxin | 0 | 82.45 | 19.56 |
| Diphenhydramine | 0 | 52.15 | 2.30 |
| Dirithromycin | 0 |  |  |
| Docetaxel | 0 |  |  |
| Dopamine | 0 |  |  |
| Doxycycline | 0 | 72.31 | 30.73 |
| Droperidol | 0 | 71.26 | 9.81 |
| Dyphylline | 0 |  |  |
| Efravirenz | 0 | 91.95 | 11.24 |
| Emtricitabine | 0 | 56.00 | 48.50 |
| Ertapenem | 0 |  |  |
| Estradiol | 0 | 87.15 | 7.48 |
| Ethylguiacol | 0 |  |  |
| Etodolac | 0 | 98.82 | 0.50 |
| Etoricoxib | 0 | 99.00 |  |
| Etravirine | 0 | 99.80 |  |
| F1594 = P-Biphenylyl-4oxo-4methyl-2 butyric acid | 0 |  |  |
| F1803 = (Orthochlorophenyl-4phenyl) hydroxyacetic acid | 0 |  |  |
| F1893 = chloro-2’-p-biphenylcarboxylic acid | 0 |  |  |
| F2006= (Orthochlorophenyl-4phenyl) acetic acid | 0 |  |  |
| Felbamate | 0 | 41.58 | 22.80 |
| Fenbufen | 0 | 99.60 |  |
| Fenoprofen | 0 | 94.82 | 6.95 |
| Floraltone | 0 |  |  |
| Flucloxacillin | 0 | 87.18 | 10.19 |
| Flufenamic acid | 0 |  |  |
| Fluorescein | 0 |  |  |
| Flurbiprofen | 0 | 97.42 | 4.52 |
| Furosemide | 0 | 72.07 | 23.35 |
| Fusidic acid | 0 | 81.34 | 15.16 |
| Ganciclovir | 0 | 38.59 | 28.37 |
| Genistein | 0 | 98.44 | 0.65 |
| Glafenic acid | 0 |  |  |
| Glibenclamide | 0 | 87.52 | 9.08 |
| Halothane | 0 |  |  |
| Hedonal | 0 |  |  |
| Heme | 0 |  |  |
| Hydrochlorothiazide | 0 | 38.23 | 13.57 |
| Hydrocortisone | 0 | 40.73 | 9.07 |
| Ibuprofen | 0 | 96.78 | 5.40 |
| Imipramine | 0 | 81.93 | 9.95 |
| Indole-3-acetic acid | 0 | 55.84 |  |
| Indole-3-acetic acid, 1-Me | 0 | 63.63 |  |
| Indole-3-acetic acid, 2-Et | 0 | 51.78 |  |
| Indole-3-acetic acid, 2-Me | 0 | 47.70 |  |
| Indole-3-acetic acid, 2-Me,5-Ome | 0 | 56.86 |  |
| Indole-3-acetic acid, 2-Pr | 0 | 58.55 |  |
| Indole-3-acetic acid, 4-Cl | 0 | 74.17 |  |
| Indole-3-acetic acid, 4-Et | 0 | 71.20 |  |
| Indole-3-acetic acid, 4-F | 0 | 66.87 |  |
| Indole-3-acetic acid, 4-Me | 0 | 58.83 |  |
| Indole-3-acetic acid, 4,5-Cl2 | 0 | 86.29 |  |
| Indole-3-acetic acid, 4,6-Cl2 | 0 | 92.35 |  |
| Indole-3-acetic acid, 4,7-Cl2 | 0 | 87.37 |  |
| Indole-3-acetic acid, 5-Br | 0 | 86.54 |  |
| Indole-3-acetic acid, 5-Bu | 0 | 91.82 |  |
| Indole-3-acetic acid, 5-Cl | 0 | 81.57 |  |
| Indole-3-acetic acid, 5-Et | 0 | 75.55 |  |
| Indole-3-acetic acid, 5-F | 0 | 64.59 |  |
| Indole-3-acetic acid, 5-Me | 0 | 62.24 |  |
| Indole-3-acetic acid, 5-Obz | 0 | 93.88 |  |
| Indole-3-acetic acid, 5-OH | 0 | 42.85 |  |
| Indole-3-acetic acid, 5-Ome | 0 | 60.49 |  |
| Indole-3-acetic acid, 5-Pr | 0 | 86.56 |  |
| Indole-3-acetic acid, 5,6-Cl2 | 0 | 96.47 |  |
| Indole-3-acetic acid, 5,7-Cl2 | 0 | 90.58 |  |
| Indole-3-acetic acid, 6-Cl | 0 | 87.94 |  |
| Indole-3-acetic acid, 6-Et | 0 | 91.71 |  |
| Indole-3-acetic acid, 6-F | 0 | 67.02 |  |
| Indole-3-acetic acid, 6-Me | 0 | 83.01 |  |
| Indole-3-acetic acid, 6,7-Cl2 | 0 | 92.48 |  |
| Indole-3-acetic acid, 7-Aza | 0 | 32.67 |  |
| Indole-3-acetic acid, 7-Cl | 0 | 80.76 |  |
| Indole-3-acetic acid, 7-F | 0 | 63.85 |  |
| Indole-3-acetic acid, 7-Me | 0 | 66.00 |  |
| Indole-3-acetic acid, Et ester | 0 | 50.00 |  |
| Indomethacin | 0 | 91.96 | 10.58 |
| Indoxyl sulfate | 0 | 99.90 |  |
| Iodipamide | 0 | 99.99 | 0.00 |
| Iopanoic acid | 0 | 99.98 |  |
| Iophenoxic acid | 0 | 100.00 | 0.00 |
| Isoxicam | 0 |  |  |
| Itanoxone | 0 | 99.15 |  |
| Itraconazole | 0 | 84.82 | 9.65 |
| JDC-108 | 0 | 99.15 |  |
| Ketoconazole | 0 | 84.91 | 8.79 |
| Ketoprofen | 0 | 86.54 | 19.19 |
| Ketorolac | 0 | 99.20 | 0.01 |
| L-Sorbose | 0 |  |  |
| Labetalol | 0 | 45.71 | 18.65 |
| Lamotrigine | 0 | 42.57 |  |
| Levocetirizine | 0 |  |  |
| Levofloxacin | 0 | 57.99 |  |
| Levothryoxine | 0 | 99.72 |  |
| Lidocaine | 0 | 56.90 | 28.05 |
| Lopinavir | 0 |  |  |
| Maltopentaose | 0 |  |  |
| Mannitol | 0 |  |  |
| Mefenamic acid | 0 |  |  |
| Meloxicam | 0 | 98.84 |  |
| Methotrexate | 0 | 37.79 | 20.69 |
| Methylprednisolone | 0 | 49.98 | 9.15 |
| Meticillin | 0 | 35.37 |  |
| Minocycline | 0 | 61.86 |  |
| Minoxidil | 0 | 33.83 | 6.85 |
| Moxalactam derivative | 0 |  |  |
| Myristic acid | 0 |  |  |
| N1-alkyl pyrimidinedione 10a | 0 | 85.90 |  |
| N1-alkyl pyrimidinedione 10b | 0 | 83.40 |  |
| N1-alkyl pyrimidinedione 10c | 0 | 99.10 |  |
| N1-alkyl pyrimidinedione 10d | 0 | 99.90 |  |
| N1-alkyl pyrimidinedione 11 | 0 | 99.30 |  |
| N1-alkyl pyrimidinedione 12 | 0 | 98.70 |  |
| N1-alkyl pyrimidinedione 13 | 0 | 98.00 |  |
| N1-alkyl pyrimidinedione 16 | 0 | 96.00 |  |
| N1-alkyl pyrimidinedione 8b | 0 |  |  |
| N1-alkyl pyrimidinedione 9 | 0 | 85.10 |  |
| Nalidixic acid | 0 | 92.92 |  |
| Naphthalene | 0 |  |  |
| Naproxen | 0 | 90.27 | 14.51 |
| Naringenin | 0 | 98.86 | 0.55 |
| Nelfinavir | 0 | 75.82 | 1.05 |
| Nelvirapine | 0 | 78.66 | 2.35 |
| Nicardipine | 0 | 87.27 | 9.48 |
| Niflumic acid | 0 |  |  |
| Nimesulide | 0 | 94.83 | 6.83 |
| Nizatidine | 0 | 30.10 | 8.41 |
| Norfloxacin | 0 | 57.99 |  |
| Nortriptylene | 0 | 79.94 | 13.93 |
| Novobiocin | 0 | 84.41 | 21.62 |
| O1-pentyl-mannose | 0 |  |  |
| Ochratoxin A | 0 |  |  |
| Ondansetron | 0 | 77.55 | 13.76 |
| Oxazepam | 0 | 88.51 | 9.49 |
| Oxprenolol | 0 | 32.08 | 13.26 |
| Oxyphenbutazone | 0 | 82.51 | 29.15 |
| Paracoxib | 0 | 99.00 |  |
| Phenacetin | 0 | 39.19 | 4.61 |
| Phenprocoumon | 0 |  |  |
| Phenylbutazone | 0 | 85.38 | 16.72 |
| Phenytoin | 0 | 68.65 | 14.50 |
| Pindolol | 0 | 32.01 | 11.83 |
| Piroxicam | 0 | 90.86 | 7.74 |
| PJ2 | 0 |  |  |
| Platinum (II) di-chloride | 0 |  |  |
| Pranoprofen | 0 |  |  |
| Prazosin | 0 | 66.09 | 13.18 |
| Prednisolone | 0 | 55.52 | 26.88 |
| Pregnenolone | 0 | 96.24 |  |
| Probenecid | 0 | 86.44 | 10.35 |
| Procainamide | 0 | 39.19 | 4.61 |
| Procaine | 0 | 46.74 | 15.86 |
| Proctolol | 0 |  |  |
| Progesterone | 0 | 79.55 |  |
| Promazine | 0 | 93.67 | 6.23 |
| Propimazine | 0 |  |  |
| Propofol | 0 |  |  |
| Propranolol | 0 | 64.67 | 4.69 |
| Propylthiouracil | 0 | 30.13 | 11.36 |
| Protriptyline | 0 | 75.18 | 9.96 |
| Proxiphylline | 0 | 32.28 | 7.36 |
| Quercetin | 0 | 99.26 | 0.36 |
| Quinidine | 0 | 65.92 | 7.64 |
| Quinine | 0 | 74.13 | 7.36 |
| Quinoline | 0 |  |  |
| R-,S-Chloroquine | 0 |  |  |
| R-,S-Warfarin | 0 | 84.18 | 20.07 |
| Ranitidine | 0 | 31.85 | 13.14 |
| Ritonavir | 0 | 71.40 | 3.14 |
| Rofecoxib | 0 | 82.00 |  |
| Ropinirole | 0 | 93.75 |  |
| Salbutamol | 0 | 29.90 |  |
| Salicylamide | 0 |  |  |
| Salmeterol | 0 | 91.10 |  |
| Salsalate | 0 |  |  |
| Saquinavir | 0 | 84.83 | 9.22 |
| Scopolamine | 0 | 31.37 |  |
| Sotalol | 0 |  |  |
| Stavudine | 0 | 83.00 | 1.41 |
| Sulfadimethoxine | 0 | 89.53 | 9.98 |
| Sulfamethizole | 0 |  |  |
| Sulfamethoxazole | 0 | 45.44 | 25.64 |
| Sulfamethoxypyridazine | 0 | 75.80 | 10.04 |
| Sulfametopyrazine | 0 | 63.53 | 6.20 |
| Sulfamoxole | 0 |  |  |
| Sulfapyridine | 0 | 26.72 | 9.15 |
| Sulfasalazine | 0 | 78.41 |  |
| Sulfinpyrazone | 0 | 88.78 | 9.73 |
| Sulfisoxazole | 0 | 83.76 | 10.19 |
| Sulindac | 0 | 92.23 | 7.55 |
| Sumatriptan | 0 | 47.12 |  |
| Suprofen | 0 | 98.91 |  |
| Suprofen ester | 0 | 98.66 |  |
| Tamoxifen | 0 | 90.64 | 8.96 |
| Tenoxicam | 0 | 99.55 |  |
| Terazosin | 0 | 40.89 |  |
| Terbinafine | 0 | 93.67 |  |
| Testosterone | 0 | 87.50 | 6.23 |
| Tetracaine | 0 | 67.63 |  |
| Tetracycline | 0 | 70.87 | 36.00 |
| Thymoquinone | 0 |  |  |
| Tipranavir | 0 |  |  |
| Tolbutamide | 0 | 81.55 | 23.97 |
| Tolmetin | 0 | 91.71 | 9.36 |
| Tramadol | 0 | 31.79 | 7.49 |
| Triflupromazine | 0 | 94.44 | 3.70 |
| Trimethoprim | 0 | 39.41 | 3.23 |
| Valdecoxib | 0 | 82.00 |  |
| Valproic acid | 0 | 97.19 |  |
| Verapamil | 0 | 69.86 | 8.33 |
| Zolmitriptan | 0 | 35.89 | 6.11 |
| Zomepirac | 0 | 96.32 | 3.78 |
| 4-hydroxylmethyl-quinolone | 1 |  |  |
| 5-fluorouracil | 1 |  |  |
| Acetaminophen | 1 | 19.48 | 8.62 |
| Acyclovir | 1 | 11.07 | 8.55 |
| Allopurinol | 1 | 0.00 |  |
| Amikacin | 1 |  |  |
| Amiloride | 1 | 24.92 | 7.58 |
| Amisulpride | 1 |  |  |
| Amoxicillin | 1 | 18.36 | 11.18 |
| Amphetamine | 1 |  |  |
| Antipyrine | 1 | 19.94 | 8.18 |
| Argatroban | 1 | 20.00 |  |
| Atenolol | 1 | 13.94 | 15.47 |
| Bethanidine | 1 |  |  |
| Bleomycin | 1 | 1.00 |  |
| Busulfan | 1 |  |  |
| Caffeine | 1 | 12.54 | 2.55 |
| Captopril | 1 | 11.60 | 16.12 |
| Cefepime | 1 | 27.43 |  |
| Cefradine | 1 | 18.65 | 5.69 |
| Cephaloridin | 1 | 15.95 |  |
| Codeine | 1 |  |  |
| Cromoglicate | 1 | 3.92 | 5.55 |
| Cyclophosphamide | 1 |  |  |
| Decitabine | 1 |  |  |
| Ebselen | 1 | 4.18 | 5.91 |
| Erythromycylamine | 1 |  |  |
| Ethosuximide | 1 | 17.39 | 0.15 |
| Etoposide | 1 | 24.45 |  |
| Fenoterol | 1 |  |  |
| Fluconazole | 1 |  |  |
| Flucytosine | 1 | 7.20 |  |
| Fludarabine phosphate | 1 |  |  |
| Fosfomycin | 1 |  |  |
| Gabapentin | 1 |  |  |
| Galantamine | 1 |  |  |
| Gemcitabine | 1 |  |  |
| Guanethidine | 1 |  |  |
| Heroin | 1 |  |  |
| Hydrocodone | 1 |  |  |
| Indinavir | 1 |  |  |
| Iohexol | 1 |  |  |
| Ioxaglic acid | 1 |  |  |
| Ioxilan | 1 |  |  |
| Ketamine | 1 |  |  |
| Levetiracetam | 1 |  |  |
| Lisinopril | 1 |  |  |
| Melphalan | 1 |  |  |
| Meptazinol | 1 |  |  |
| Mercaptopurine | 1 |  |  |
| Metformin | 1 |  |  |
| Methimazole | 1 |  |  |
| Methyldopa | 1 |  |  |
| Metoprolol | 1 | 24.70 | 13.01 |
| Morphine | 1 |  |  |
| Moxisylate | 1 | 28.86 |  |
| Nadolol | 1 | 20.45 | 14.71 |
| Nelarabine | 1 |  |  |
| Nicotine | 1 |  |  |
| Oseltamivir | 1 |  |  |
| Penicillin V | 1 | 16.96 |  |
| Pentostatin | 1 |  |  |
| Practolol | 1 | 13.63 |  |
| Pramipexole | 1 |  |  |
| Rizatriptan | 1 |  |  |
| Sotalol | 1 | 29.04 | 17.50 |
| Spiramycin | 1 |  |  |
| Tenofovir | 1 |  |  |
| Thiotepa | 1 |  |  |
| Timolol | 1 | 24.93 | 9.81 |
| Topiramate | 1 |  |  |
| Topotecan | 1 |  |  |
| Tranexamic acid | 1 |  |  |
| Venlafaxine | 1 |  |  |
| Zalcitabine | 1 | 63.00 |  |
| Zanamivir | 1 |  |  |
| Zidovudine | 1 | 22.97 | 10.26 |
| Antrafenine | 2 |  | |
| Aspirin | 2 | 60.23 | 40.28 |
| azithromicin | 2 |  |  |
| cefuroxime | 2 | 39.17 | 32.08 |
| lamivudine | 2 | 84.00 |  |
| Linezolid | 2 |  |  |
| L-tryptophan | 2 | 73.37 | 39.66 |
| neostigmine | 2 | 62.30 | 28.17 |
| salicylic acid | 2 | 66.75 | 34.29 |
| sulfaphenazole | 2 | 68.66 | 43.15 |
| Tolazamide | 2 | 79.91 | 34.92 |

All ligands included in the study, with their status as an HSA binder described as 0=binder, 1=weak/non-binder, or 2=conflicting reports. Their average %HSA score and SD if applicable are also shown.
