# Supplementary material for: A Structure-Based Model for Predicting Serum Albumin Binding
Source: PLoS One. 2014 Apr 1;9(4):e93323. doi: 10.1371/journal.pone.0093323 (PMC3972100; doi:10.1371/journal.pone.0093323)
Supplement: Table S2 — Results from model applied to four literature sets. Literature data sets for HSA binding, derived from Colmenarejo, Kratchowil, Valko, and Zsila, as well as all ligands with binder/nonbinder status. ROC plot AUCs are analyzed as a metric for success in predicting binding to HSA using QPlogPo/w; Schrödinger's metric for HSA binding, QPlogKhsa, which was developed using the Colmenarejo set and overlaps with the Valko set; rigid receptor docking; or IFD. The Colmenarejo set includes high-scoring false positives cromolyn, ebselen, and pencillin V. (DOCX) [file pone.0093323.s011.docx]

**SI Table S2**. **Results from model applied to four literature sets.**

| **Docking method** | **Sites Docked Against** | **PDB structures** | **Colmenarejo** | **Kratchowil** | **Valko** | **Zsila** | **All** |
| --- | --- | --- | --- | --- | --- | --- | --- |
| None: QPlogP_o/w_ | | | 0.82 | 0.83 | 0.89 | 0.81 | 0.78 |
| None: QPlogKhsa | | | 0.88 | 0.77 | 0.90 | 0.72 | 0.75 |
| Rigid | Sites I and II | 10 structures | 0.68 | 0.64 | 0.72 | 0.71 | 0.69 |
| IFD | Sites I and II | 10 structures | 0.68 | 0.78 | 0.76 | 0.73 | 0.70 |
| IFD | Sites I and II | 2BXP and 1N5U | 0.71 | 0.74 | 0.76 | 0.72 | 0.70 |
| IFD | Sites I and II (with fatty acid) | 2BXP and 1N5U | 0.62 | 0.76 | 0.72 | 0.83 | 0.75 |
| IFD + QPlogP_o/w_ | Sites I and II (with fatty acid) | 2BXP and 1N5U | 0.79 | 0.87 | 0.90 | 0.87 | 0.83 |

Literature data sets for HSA binding, derived from Colmenarejo, Kratchowil, Valko, and Zsila, as well as all ligands with binder/nonbinder status. ROC plot AUCs are analyzed as a metric for success in predicting binding to HSA using *QPlogP_o/w_*; Schrödinger’s metric for HSA binding, *QPlogKhsa*, which was developed using the Colmenarejo set and overlaps with the Valko set; rigid receptor docking; or IFD. The Colmenarejo set includes high-scoring false positives cromolyn, ebselen, and pencillin V.
