# Supplementary material for: A Structure-Based Model for Predicting Serum Albumin Binding
Source: PLoS One. 2014 Apr 1;9(4):e93323. doi: 10.1371/journal.pone.0093323 (PMC3972100; doi:10.1371/journal.pone.0093323)
Supplement: Table S3 — Refinement scores for PDB structures. PrimeX refinement scores for the two PDB structures used in the final, combined, model. (DOCX) [file pone.0093323.s012.docx]

**SI Table S3**. **Refinement scores for PDB structures.**

| **PDBID** | **Original R-work** | **Original R-free** | **Refined R-work** | **Refined R-free** |
| --- | --- | --- | --- | --- |
| **1N5U** | 0.235 | 0.280 | 0.231 | 0.279 |
| **2BXP** | 0.211 | 0.250 | 0.199 | 0.250 |

PrimeX refinement scores for the two PDB structures used in the final, combined, model.
